# Supplementary material for: Patients’ initial steps to cancer diagnosis in Denmark, England and Sweden: what can a qualitative, cross-country comparison of narrative interviews tell us about potentially modifiable factors?
Source: BMJ Open. 2017 Nov 19;7(11):e018210. doi: 10.1136/bmjopen-2017-018210 (PMC5702025; doi:10.1136/bmjopen-2017-018210)
Supplement: Author's manuscript [file bmjopen-2017-018210.draft_revisions.pdf]

BMJ Open is committed to open peer review. As part of this commitment we make the peer review history of every article we publish publicly available.

When an article is published we post the peer reviewers' comments and the authors' responses online. We also post the versions of the paper that were used during peer review. These are the versions that the peer review comments apply to.

The versions of the paper that follow are the versions that were submitted during the peer review process. They are not the versions of record or the final published versions. They should not be cited or distributed as the published version of this manuscript.

BMJ Open is an open access journal and the full, final, typeset and author-corrected version of record of the manuscript is available on our site with no access controls, subscription charges or pay-per-view fees (http://bmjopen.bmj.com).

If you have any questions on BMJ Open's open peer review process please email editorial.bmjopen@bmj.com

BMJ Open

Initial steps to cancer diagnosis in Denmark, England, and Sweden: what can a qualitative, cross country comparison of narrative interviews tell us about potentially modifiable factors?

Journal:	*BMJ Open*
Manuscript ID	bmjopen-2017-018210
Article Type:	Research
Date Submitted by the Author:	15-Jun-2017
Complete List of Authors:	MacArtney, John; University of Oxford, Health Experiences Research Group, Nuffield Department of Primary Care Health Sciences<br>Malmström, Marlene; Lund University and Region Skåne, The Institute for Palliative Care; Lund University, Skane University Hospital, Department of Clinical Sciences Lund, Surgery<br>Overgaard Nielsen, Trine; Aarhus Universitet, Research Centre for Cancer Diagnosis in Primary Care, Research Unit for General Practice, Department of Public Health<br>Evans, Julie; University of Oxford, Health Experiences Research Group, Nuffield Department of Primary Care Health Sciences<br>Bernhardson, Britt-Marie; Karolinska Institutet, Department of Learning, Informatics, Management and Ethics<br>Hajdarevic, Senada; Umeå University, Department of Nursing<br>Chapple, Alison; University of Oxford, Health Experiences Research Group, Nuffield Department of Primary Care Health Sciences<br>Eriksson, Lars; Karolinska Institutet, Department of Learning, Informatics, Management and Ethics; City, University of London, School of Health Sciences<br>Locock, Louise; University of Oxford, Health Experiences Research Group, Nuffield Department of Primary Care Health Sciences<br>Rasmussen, Birgit; Lund University and Region Skåne, The Institute for Palliative Care; Lunds Universitet, Department of Health Sciences<br>Vedsted, Peter; Aarhus Universitet, Research Centre for Cancer Diagnosis in Primary Care, Research Unit for General Practice, Department of Public Health; Aarhus Universitet, Department of Clinical Medicine<br>Tishelman, Carol; Karolinska Institutet, Medical Management Centre, LIME; Karolinska Universitetssjukhuset, Center for Innovation<br>Andersen, Rikke; University of Aarhus, Research Centre for Cancer Diagnosis in Primary Care, Research Unit for General Practice, Department of Public Health<br>Ziebland, Sue; University of Oxford, Health Experiences Research Group, Nuffield Department of Primary Care Health Sciences
**Primary Subject Heading**:	Health services research
Secondary Subject Heading:	Qualitative research, General practice / Family practice
Keywords:	PRIMARY CARE, QUALITATIVE RESEARCH, International health services <

For peer review only - http://bmjopen.bmj.com/site/about/guidelines.xhtml

	HEALTH SERVICES ADMINISTRATION & MANAGEMENT

For peer review only

Title: Initial steps to cancer diagnosis in Denmark, England, and Sweden: what can a qualitative, cross country comparison of narrative interviews tell us about potentially modifiable factors?

List of authors

John I MacArtney[a], Senior Researcher.

Marlene Malmström[b, c], Senior Researcher.

Trine L Overgaard Nielsen[d], Research Assistant.

Julie Evans[a], Senior Researcher.

Britt-Marie Bernhardson[e], Senior Researcher.

Senada Hajdarevic[f], Senior Lecturer.

Alison Chapple [a], Senior Researcher.

Lars E. Eriksson[e, g, h], Associate Professor.

Louise Locock[a], Associate Professor.

Birgit H Rasmussen[b, i], Professor.

Peter Vedsted[d, j], Professor.

Carol Tishelman[e, k], Professor.

Rikke Sand Andersen[d], Associate Professor.

Sue Ziebland[a], Professor.

[a] Health Experiences Research Group, Nuffield Department of Primary Care Health Sciences, University of Oxford, UK

[b]The Institute for Palliative Care, Lund University and Region Skåne, Sweden.

[c]Lund University, Skane University Hospital, Department of Clinical Sciences Lund, Surgery, Lund, Sweden

For peer review only

[d]Research Centre for Cancer Diagnosis in Primary Care, Research Unit of General Practice, Aarhus University, Denmark

[e]Department of Learning, Informatics, Management and Ethics, Karolinska Institutet, Sweden

[f]Department of Nursing, Umeå University, Sweden

[g]Department of Infectious Diseases, Karolinska University Hospital, Stockholm, Sweden

[h]School of Health Sciences, City, University of London, United Kingdom

[i]Lund University, Department of Health Sciences, Lund, Sweden

[j]University Clinic for Innovative Patient Pathways, Silkeborg Hospital, Department of Clinical Medicine, Aarhus University, Denmark.

[k]Center for Innovation, Karolinska University Hospital, Stockholm Sweden

Corresponding author:

John I MacArtney

Nuffield Department of Primary Care Health Sciences

University of Oxford

Radcliffe Observatory Quarter

Woodstock Road

Oxford

OX2 6GG

john.macartney@phc.ox.ac.uk

For peer review only

Abstract

Objectives

To illuminate patterns observed in International Cancer Benchmarking Programme studies by extending understanding of the various influences on presentation and referral with cancer symptoms.

Design

Cross-country comparison of Denmark, England, and Sweden with qualitative analysis of in-depth interview accounts of the pre-diagnostic process in lung or bowel cancer.

Participants

155 women and men, aged between 35 and 86 years old, diagnosed with lung or bowel cancer in the six months before interview.

Setting

Participants recruited through primary and secondary care, social media, support groups and word-of-mouth. Interviews collected by social scientists or nurse researchers during 2015, mainly in participants' homes.

Results

Participants reported difficulties in interpreting diffuse bodily sensations and symptoms and deciding when to consult. There were examples of swift referrals by primary care professionals in all three countries. In all countries participants described difficulty deciding if and when to consult, highlighting concerns about access to GP appointments and overstretched primary care services, although this appears less prominent in the Swedish data.

For peer review only

It was not unusual for there to be more than one consultation before referral and we noted two distinct patterns of repeated consultation: (1) Situations where the participant left the PCP consultation with a plan of action about what should happen next. (2) Participants were unclear about under which conditions to return to the doctors. This second pattern sometimes extended over many weeks during which patients described uncertainty, and sometimes frustration, about if and when they should return and whether there were any other feasible investigations. The latter pattern appeared more evident in the interviews in England and Denmark than Sweden.

Conclusion

If clear action plans, as part of safety netting, were routinely used in primary care consultations then uncertainty, false reassurance, and the inefficiency and distress of multiple consultations could be reduced.

For peer review only

Strengths and limitations of this study:

- This study provides a social science informed, qualitative cross-country comparison of 155 in-depth interviews with bowel and lung cancer patients recruited within six months of a diagnosis in Sweden, Denmark and England.
- The methods we use helped to provide insight into why and how potentially modifiable factors identified by the International Cancer Benchmarking Project - including response to symptom experiences, differences in willingness to consult, how people negotiate access to healthcare, and what happens during consultations - affect the time to diagnosis in a primary care setting.
- Our analysis was able to show how patients' narratives suggested differences between the three countries in the clarity and communication of action plans at the end of PCP consultations.
- The study was limited to patient interviews and no 'first hand' observational data were available to compare accounts.

For peer review only

Introduction

Background

No single factor is likely to explain why differences in cancer outcomes persist between high-income countries with universal health coverage. The International Cancer Benchmarking Partnership (ICBP), which compares cancer registry data and cross country surveys, has explored several hypotheses about why variations occur. These studies have shown, for example, that patterns in public knowledge about cancer awareness and beliefs were not clearly associated with variations in survival across countries.[1] The ICBP studies have also drawn attention to different patterns in public willingness to consult a Primary Care professional (PCP), with patients in the UK reporting particular concern 'not to waste the doctor's time'.[1] There are also cross country differences in general practitioners (GPs) expressed willingness to refer a patient with suspicious symptoms at their first presentation and PCPs have reported that investigations such as computerised tomography (CT) and Magnetic resonance imaging (MRI) were harder to access and results took longer in England and Denmark, than in Sweden.[2] There seems to be a relationship between smoking behaviour and willingness to consult: in separate, recent, studies both English [3] and Danish smokers [4] have been shown to be less likely than non-smokers to consult with red flag lung cancer symptoms. It has been suggested that this is related to the shame, blame and stigma associated with lung cancer as a smoking related disease.[5] Socio-economic deprivation, inequalities in service provision and regional differences in expectations about the likely consequences of seeking care further contribute to a complicated picture.

Denmark, England, and Sweden (all ICBP participants) were selected for this comparison because survival for lung and bowel cancer between 1995 and 2007 were persistently higher in Sweden than in Denmark or England, particularly in the first year after diagnosis.[6]

For peer review only - http://bmjopen.bmj.com/site/about/guidelines.xhtml

Analyses have drawn attention to between country differences in stage at diagnosis and access to good care, diagnostics and screening.[7] Late presentation and long diagnostic intervals are potentially modifiable contributory factors to poor survival.[6-11] Cross country comparative research can suggest routes for service redesign as well as hypotheses for further research.[12] Earlier diagnosis has become a key healthcare research and policy target[13 14] and a focus of the National Awareness and Early Diagnosis Initiative (NAEDI) in England.[15] Potentially modifiable factors affecting the time to diagnosis include public response to symptom experiences, differences in willingness to consult, how people negotiate access to healthcare, what happens during consultations, and access to diagnostic tests.[1 11 16-19] While surveys and cancer registry data can provide excellent high level comparative data showing patterns of association, in-depth qualitative research is needed to help illuminate why and how observed variations may occur.[20 21] We therefore designed a study to contribute a qualitative cross-country comparative analysis of narrative interviews with patients recently diagnosed with lung or bowel cancer in Denmark, England, and Sweden. In this paper we ask what might explain some of the variations identified in the ICBP.

The healthcare systems in Denmark, England and Sweden.

All three countries have primarily tax-based health systems. However, in Sweden patient co-payments vary from no payment to 300SEK per primary care visit, depending on county council, up to a total of ~1,100SEK (£100) per year.[22] No co-payments are made in England and Denmark, although there are prescription charges in all three countries. In Sweden two-thirds of GPs are publically employed, whereas in Denmark and England most are self-employed.[22] Sweden spends over 20% more on healthcare per head of population than England.[23] Contact with primary care varies between the three countries: Swedish patients consult PCPs least frequently (2.9 per annum) followed by Denmark (4.7 per annum) and UK

For peer review only - http://bmjopen.bmj.com/site/about/guidelines.xhtml

(5.0 per annum, which includes telephone consultations).[24] In 2015 England withdrew guidance stipulating access for urgent appointments within 48 hours.[22] In some settings in Sweden, a nurse is the first person a patient speaks to when seeking an appointment.[22] Most first contact in Denmark and England is with a receptionist, who may pass the patient to a nurse to be triaged. In Denmark some receptionists have been trained to recognise potentially serious symptoms.[22] GPs have a central role in making referrals to lung and colorectal specialists in all three countries [22] The national cancer plan in Sweden is regionally administered and focused on secondary care[25], whereas in England and Denmark specific clinical guidance and targets for PCPs are also included.[22] England (implemented 2006) and Denmark (implemented 2014) have national cancer screening programmes for bowel cancer, while in Sweden bowel screening is recommended for 60-74 year olds but the programme varies by region. Denmark has increased rapid access clinics and access to investigations, while in England referral management systems have been interspersed between the GP and secondary care.[22 26]

Methods

Sampling and recruitment

We interviewed 155 people during 2015 who were within six months of a diagnosis of lung or bowel cancer in the three countries. Purposive sampling[27] within each country was used to achieve gender balance and variation across age, urban and rural locations, and pathway to diagnosis. An experienced sociologist in England (JM) and an anthropologist in Denmark (TON) recruited and conducted all the interviews in their countries, while three experienced nurse researchers collaborated in different regions of Sweden (BMB, SH, and MM). Participants were initially approached by treating clinicians in hospital clinics in all three countries, which was supplemented in England and Denmark with recruitment from support

For peer review only

For peer review only - http://bmjopen.bmj.com/site/about/guidelines.xhtml

groups, social media, and word-of-mouth. All potential participants were provided with an information sheet that included the rationale for the study and an explanation of what would be involved if they consented to take part in the study. Data saturation[28] was judged to have been reached in the analytic categories for all three countries before recruitment closed. Participant ages ranged from 35 to 86 years.

Data collection

Interviews took place in participants' homes, unless they preferred another location. One-to-one interviews were preferred, but a small number of participants requested a non-participating family member to be present. Interviews started with an open ended question: "Could you start by telling me, in your own words and in as much detail as you want, about everything that has happened since you first started to suspect there might be a problem with your health?" Interviews lasted between 45 minutes to 1.5 hours. During the interview the researchers used a semi-structured topic guide based on social science theories, and cancer research literature, including factors related to the diagnostic interval.[5 29-34] The research team had extensive discussions about the topics to ensure comparable data were collected. Interviews were audio recorded and transcribed verbatim.

Analysis

Monthly teleconferences with the field research team (all of whom had a high level of spoken and written English) were held throughout the recruitment, data collection and analysis phases. Interview accounts were analysed for narrative themes that structured participant experiences. To do this, in each country interview transcripts were imported into specialist computer software (NVivo 10) for organising textual data and were coded by the interviewing researchers. The three research teams conducted separate thematic analysis with

For peer review only

their own data using a coding frame developed through discussion in the teleconferences and based on the (anticipated) themes from the topic guide and on emergent themes.[35] One two-day and one four-day analysis and writing workshops were held with the research teams from all three countries. Anticipated issues drew on existing knowledge and theoretical insights.[5, 16-19, 29, 32-34, 36] Emerging analyses were iteratively tested within and between datasets. Allocations between the analysis categories (including identifying participants who described leaving the PCP consultation with an understanding of what should happen next and those who reported uncertainty about what to do) was discussed among the field researchers. Direct quotes from the interviews (translated into English by the bi-lingual researchers) are used to illustrate the results. Analysis and findings are presented in accordance with relevant COREQ[37] criteria for reporting qualitative data and are consistent with interpretive approaches to analysis.[38]

Patient involvement

Public and Patient Involvement (PPI) was conducted in accordance with good practice in each country. Representatives in England with experience of bowel or lung cancer were involved in preparing the funding application, the study design and the multidisciplinary advisory group. PPI members were invited to comment on the funding application and the draft interview topic guide, and advised on recruitment strategies, including the patient information sheets. A group of three English PPI members met with the research team to look at a subset of the English data. Drafts of study papers have been circulated to the advisory panel. Summaries of the main study findings, prepared for a stakeholder final event in 2017, will be circulated to all study participants who expressed interest in seeing the results.

For peer review only - http://bmjopen.bmj.com/site/about/guidelines.xhtml

Results

We compared the three sets of interviews to consider the following potentially modifiable steps, drawn from the literature, on the pre-diagnosis pathway: people's initial responses to bodily sensations and the transformation of these into symptoms justifying medical advice; accounts of accessing medical help; what happened in the consultation; and, planning for follow-up.

Awareness and responses to signs and symptoms

The first potentially modifiable factors are the type of sensations and bodily changes people considered to justify seeking help. Participants in all three countries described considerable uncertainty about the possible implications of bodily sensations and when or if they should be considered symptoms warranting medical advice, especially if they felt well. For example, apart from some blood in his stools a participant in England said he had been "fit as anything and not feeling ill at all" (England-BC18). Even those who looked up their symptoms and risk factors online were reassured if they found that relatively few items on the checklists applied to them. People reasoned that they must be low risk if they felt they had a good diet, were physically active, non-smokers (or long term ex-smokers), generally fit, relatively young, or without a family history of cancer. This reasoning suggested that some patients expected there to be a linear relationship between healthy input and outcomes. For example, a participant in Denmark who acknowledged "[I] smoke and [drink] alcohol and [eat] too much meat…" went on to suggest that other behaviours offered some protection from the profile of a typical cancer patient:

> Denmark-BC01: I have read about this, I'm very much into organic food. All the things you say can cause cancer… you don't know […]. I have at least lived a healthy life with vegetables everyday…and fruit, so maybe I'm not the typical cancer patient.

For peer review only - http://bmjopen.bmj.com/site/about/guidelines.xhtml

Persistent coughs, pains, stomach aches, changes in bowel habits, bleeding or constipation were attributed to everyday causes or to more familiar diseases such as influenza, pneumonia, Irritable Bowel Syndrome (IBS) or hemorrhoids.[30] People with lung cancer, especially those who smoked cigarettes, reasoned that a persistent cough was a ‘normal’ bodily response to smoking. Thus, even those who are aware that they have risk factors, or suspicious symptoms, may conclude that overall their risk is low and not seek medical help. Consistent with Zola’s classic work on triggers to the consultation, when people *did* consult this was usually because the symptoms: interfered with daily life; became evident to others who encouraged them to consult; or, exceeded a self-imposed time limit[31], rather than because they suspected that they might have cancer.

Access to primary care

The second potentially modifiable factor is whether the participant felt able to access primary care. There were some differences apparent in accounts from the three countries. In England, people talked about long waiting times for ‘non-urgent’ appointments or (echoing ICBP findings) said they were reluctant to trouble busy GPs with potentially minor illnesses that might be treatable with over-the counter remedies.[1] A participant in England, later diagnosed with bowel cancer, had concluded, “It’s haemorrhoids, leave it. Doctors are busy with other things” (England-BC20). Another participant in England with lung cancer explained why she hadn’t consulted with a cough:

> England-LC12: When you hear that the GPs are so busy and being bothered by, you know, minor illnesses it makes you reluctant to go with something that you think [is minor] . . . I was, well, “What can the doctor do? They won’t want to give you

For peer review only

> antibiotics for a virus." That was what I, I thought. And I just didn't want to really bother the doctor.

A participant in Denmark similarly reflected upon an implication of GP's time pressures:

> Denmark-LC15: The system is not always that easy. First you have to convince the secretary, that you need an appointment, right. That is what happens when they are too busy.

For peer review only

In contrast, we were struck that accounts in Sweden rarely included detailed justifications for using the doctor's time, nor did they suggest that the onus was on the participant to determine whether the matter was serious, or urgent. For example, a participant in Sweden diagnosed with bowel cancer said:

> Sweden-BC051: It began like this, that I found a little blood ... I can say in the stool, very little . . . And I tend to ignore such things ... but this was just strange enough, so I thought, "no, I've got to go to the health centre and hear what it is". And so I did.

Another participant in Sweden with lung cancer said, "It's better to go one time too many than one too few" (Sweden-LC102). This sentiment was not expressed in any of our interviews in England.

During the consultation

The third potentially modifiable factor is what happens when the patient presents with symptoms. Seeking healthcare is not just a matter of making an appointment, but also of being able to present one's problem and be heard.[14] In all three countries there were examples of a single consultation and a prompt referral. A participant in England said,

For peer review only - http://bmjopen.bmj.com/site/about/guidelines.xhtml

> England-BC18: Our local GP was brilliant, she said, "I'm not gonna do the kind of normal give you this stool sample packet, all that kinda stuff. I'm gonna hotline you straight to [hospital] down the road there for an endoscopy. Skip all the intermediate stuff and go straight into endoscopy", which was a fabulous decision, if you think of it.

Similarly a participant in Denmark explained,

> Denmark-BC13: The stomach pains had been going on for a while, but one day I just could not stand it anymore and drove straight down to my doctor's office. My doctor examined it and immediately said: "I will send you off to the hospital". And I was [sent off] in the afternoon. That was quick.

A participant in Sweden with bowel cancer recalled the GP saying, "No, I cannot see anything, but for safety's sake I will send you to a specialist […] so they get a closer look at it" (Sweden-BC051).

There were also examples of participants in all three countries who told us that they had returned to the doctors over periods of weeks or even months, before they were referred for further investigations. Some participants in England and Denmark diagnosed with lung cancer said they had been repeatedly treated with antibiotics for chest infections. Participants diagnosed with bowel cancer from all three countries described inconclusive consultations for presumed constipation, haemorrhoids, IBS, Crohn's, or psychological causes; however in our data this appeared more often in interviews from Denmark and England. This example from England typifies this:

For peer review only

England-BC09: . . . each time it was treated obviously as constipation. There was never even any mention of anything else. . . He's (the GP) a very, "I can do it. I can fix it. I can do it". But each time I come away, it was so sort of disheartening, because it seemed to be about what he could do and not actually helping the situation. But I did have, I had all sorts of laxatives from him. It's as if like we just keep trying to do this, but I was in agony each time I took it. I did explain that each time. . . . But, yeah, nothing really. It was more like the repetitive sort of appointment.

Making plans for follow-up

The fourth potentially modifiable factor is what happens after those consultations which do not include a referral. We looked in detail at the accounts of those participants who reported two or more presentations before referral and noted two different types of experience, depending on whether or not the patient reported leaving the consultation knowing what to do next.

Repeated consultation(s) with patient awareness of next steps

First, there were participants in all three countries who described leaving the consultation with an understanding about what to expect, including how long to wait for symptoms to abate, what should prompt them to return, and what would happen after that. For example this participant in Sweden said,

Sweden-LC129: And then the [GP] said, "No it's nothing. Your lungs sound very healthy, but you've had this cough for the last eight weeks and if it does not disappear in another week, then please call us. Then we'll see."

For peer review only

The participants in this ‘planned’ group had also known what would be likely to happen if and when they did return; all three countries included examples of people who knew that further treatments or investigations would be needed if the symptoms did not resolve:

> England-LC17: And he [GP] prescribed two weeks antibiotics. And then he said that after that, if it didn’t make any difference then he might prescribe omeprazole and maybe then a chest x-ray.

Follow-up advice can be reinforced by practice staff when delivering a normal test result in the course of their diagnostic investigations.[39] For example a participant in Sweden reported that the practice nurse said, “if [it] gets worse – for example if you lose weight – contact us directly” (Sweden-BC055).

Repeat consultation(s) with patient uncertainty about next steps

Second, there were participants who were unsure what would or *should* happen next, such as England-BC09 quoted above who described a “disheartening” and “repetitive sort of appointment”. These participants included those who worried that it was not appropriate to return to the doctor with the same symptoms and those who had been reassured at the initial consultation. The following participant in Denmark had requested a colonoscopy after many months of unusual symptoms and several investigations for other possible causes.

> Denmark-BC05: I can see now that I had these symptoms for three years. They [the doctors] thought that it was only the myalgia. But it probably was the colon cancer, even though I did not have the usual symptoms, you know. No bleeding, or diarrhoea or constipation. Just this strange heaviness in my lower parts. So I had an ultrasound of my bladder and kidneys, an x-ray of my lumbar area… And then I finally asked for

For peer review only

a colonscopy. My doctor asked me if I was sure… of course I was… but I had not thought of cancer.

In these accounts people had been unsure what to expect and left without a plan about what to do next. Unsurprisingly, people who had presented on two, three or more occasions described some frustration. In retrospect, some wondered if the PCP had decided that a "test of time" was the best course of action, without sharing their reasoning.[40]

England-BC03: And the simple thing and I agree the most obvious thing was, you know, piles and irritable bowel syndrome . . . I guess they, the thought was, "Well we'll send her away with that for now and if doesn't work, she's bound to come back."

Accounts of repeated consultations, without an action plan communicated to the patient, featured more in the interviews with patients in England and Denmark than in Sweden. Patients may welcome reassurance from their PCP about their suspicious symptoms, and this may not be a problem if the practitioner takes responsibility for following up the patient as part of safety netting – the process of communicating with patient what to expect, documenting any action plans, and following the patient up as agreed.[41] A Swedish lung cancer patient explained that the GP had telephoned to see 'how she was' after a gastroscopy and made an urgent referral:

Sweden-LC123 I got a referral to ultrasound of my thyroid and upper gastro. And then they discovered in a gastroscopy that I had oesophagitis . . . [a week later the GP] called to ask how I was. I said, "now it is bad", I said. "I think I have to go to the hospital urgently as it is now, it's ... I cannot breathe". "Come here", she said, "and we'll make an urgent referral".

For peer review only - http://bmjopen.bmj.com/site/about/guidelines.xhtml

Discussion

Principal findings

Our international qualitative comparison has considered four potentially modifiable features of the pre-diagnostic experience of lung and bowel cancer patients. Across the countries there are many similarities in how symptoms were experienced and in awareness of what the symptoms might mean. Our analysis suggests differences between countries in people's accounts of their willingness to consult and re-consult, their descriptions of what happened during consultations and, for those who did not receive an immediate referral, their confusion or clarity about what they should look for, when to return and what to expect. Reluctance and practical barriers to consulting the GP, and uncertainty about whether and when to re-attend after an initial consultation, appeared more evident in interviews in Denmark and England. This indicates the benefit of communication of clear action plans to patients, including information on symptom development, and other safety netting approaches,[42] which could well be developed more. PCPs action plans might include a 'test of time', yet this reasoning should be clearly communicated to the patient. This could also mean that patients would receive more timely referrals, and avoid the repeated visits to primary care which featured particularly in English and Danish interviews.[40]

Strengths and weaknesses of the study

The study design – a cross-cultural comparison using in-depth qualitative interview – is the first of its kind to study the route to cancer diagnosis. Our findings suggest potentially modifiable factors that could improve timely diagnosis of cancer in all three countries, although the scope for improvement may be greater in England and Denmark.

For peer review only

This qualitative study was designed to illuminate findings from the ICBP studies and is intended to be indicative rather than conclusive. Findings extend observations from other research that highlights the centrality of the primary care encounter in pre-diagnostic experiences. We chose lung and bowel cancer because they affect both genders, are the two most common causes of death from cancer across Europe,[43] their symptomatology is often diffuse or vague, and these cancers are often prone to late stage diagnosis. All interviews were collected within approximately six months of diagnosis. People who agreed to take part in our research may have been less unwell than those who did not. All participants were, or had been, under specialist cancer care, which may have affected the way they reflected upon the time and events that passed before their diagnosis.[38] However, this is a comparative study and there is no reason to suspect that any ‘recall bias’ (as epidemiologists would see it) might function differently in the three countries. Each data set was analysed by the country specific team, thus benefitting from familiarity with healthcare context, language and culture. These country specific analyses were shared as part of a careful collaborative process, involving workshops, teleconferences and several drafts and further targeted analyses.

Strengths and weaknesses in relation to other studies

This study was designed to explore whether a cross-country comparison of qualitative interviews could help to illuminate findings from the ICBP survival analyses and surveys. While the high level comparisons of large quantitative data bases and surveys provide invaluable information, qualitative studies can suggest why these patterns may occur and identify avenues for service redesign, some of which could be implemented without further research. We did not observe the consultations described by the patients and it is possible that the PCPs involved did try to communicate an action plan, even if it did not feature in the patient’s account. Again, we have no reason to suspect that variations in recall would differ

For peer review only

between the countries. It is well known that public knowledge of cancer symptoms is insufficient to prompt people to seek help, as recently demonstrated in ICBP surveys.[1] Our work was guided by insights from the social and healthcare sciences into the complexity inherent in decisions to consult. These are influenced by people's ideas about their own candidacy, related to family history and health behaviours, as well as their access to care.[44 45] Evans et al have shown that patients, and their doctors, may be falsely reassured if a symptom comes and goes, reasoning that intermittent symptoms are unlikely to be serious.[33] Renzi and colleagues have drawn attention to the potential negative consequences of reassurance through an 'all-clear' result from investigations, which could be given with or without an encouragement to return.[39]

This study provides further insight into how cancer symptoms are managed in primary care, in particular how delays may be compounded when a patient is not referred promptly and no clear plan is communicated about the circumstances in which they should return for further consultation or investigations.[2 46] Current research is exploring how and why planning, communicating action plans to patients, and following-up are operationalised as part of safety netting in English primary care and what approaches are acceptable to both GPs and patients.[41]

Implications for clinicians and policy makers

There are several important differences between these three countries, for example in access to secondary care, in spending on health services and in the numbers of licensed practitioners. The health system in England has less money, fewer doctors and nurses and fewer hospital beds then Sweden or Denmark.[22-24] These factors may yet prove critical in limiting improvements in cancer survival. Patients in Sweden consult their GP less often than patients

For peer review only - http://bmjopen.bmj.com/site/about/guidelines.xhtml

in England or Denmark, but for longer.[24] Shorter consultations may not be efficient – patients in England consult their GPs for nearly one hour per year, yet spread over five appointments while, on average, patients in Sweden visit primary care twice a year, but for a mean of 20 minutes per appointment.

Through our international comparison we have been able to identify opportunities for pathway and service improvement that could promote earlier stage diagnosis. The perception of systemic barriers (e.g. access to PCP appointments) may prevent patients seeking timely help, even in the presence of potentially alarming symptoms (e.g. blood in stools or a cough that lasts for three weeks). While awareness of symptoms may be important, there is a limit to how much effect increasing public awareness could have in settings where access is limited and people are uncertain when to consult, or re-consult.[32 47]

The importance of safety netting was emphasised in the 2015 guidance for suspected cancer from the UK National Institute for Health and Care Excellence[48] and a 2011 Delphi study of British GPs and primary care researchers led to publication of suggestions for how to make safety netting more effective.[41] In Denmark, direct access to investigation from general practice, with the aim to lower the GPs' threshold for referring and lengthy 'wait-and-see' pathways, is being investigated along with a focus on safety-netting. Our findings suggest that clearly communicated action plans at the end of *any* primary care consultation could be encouraged, without the need for further research. This would be likely to improve the patient's experience and may also reduce demand for repeated appointments and diagnostic delay.

For peer review only

Unanswered questions/need for further research

Research is needed into ways of redesigning primary care services in all three countries to reduce (perceived) barriers to consultation. NICE has recommended that British PCPs employ safety-netting to reduce delays in the diagnosis of cancer, but there has been little research to understand what constitutes best practice. Further research might address how a "time will tell" approach that includes clear planning affects the number of follow-up visits and cancer stage at diagnosis. Cross country differences in referral and follow up practices in lung cancer and bowel cancer patients could be investigated further, especially in the light of recent studies suggesting that smokers (in Denmark and England) may be less likely to consult. Is the same pattern apparent in Sweden – and if not, why not?

Conclusion

If all primary care consultations concluded with a clear description of what should happen next and the circumstances in which the patient should return, this might avert some of the uncertainty and repeated consultations that were described in these interviews. We conclude that there is an opportunity for clearer communication and better follow-up strategies in primary care – neither of which will put additional pressure on specialist services, yet hold the potential to reduce diagnostic delays with few additional resources.

For peer review only

Other BMJ requirements

Acknowledgements

We are extremely grateful to the people who took part in this research in all three countries, and to the study advisory panel, including patient and public representatives, who helped design the study and provided comments on an earlier draft of this manuscript. We also acknowledge the support of the National Institute for Health Research, through the Clinical Research Network, who helped recruit patients into the English arm of the study. We would also like to thank all those who helped to recruit participants: In England we would like to thank the NHS Hospital Trusts that assisted with this study and Patients Active in Research: Thames Valley, along with the following charities who posted links or circulated our details on social media: Beating Bowel Cancer, Bowel Cancer UK, Roy Castle Lung Cancer Foundation, British Lung Foundation, and healthtalk.org. In Denmark we want to thank the Lung Cancer Patient organisation, as well as the Colon Cancer Patient organisation for assisting us in recruiting patients. We also wish to thank the local support groups of the national Danish Cancer Society, as well as the local oncology departments in various regions of Denmark. In Sweden we would like to thank the nurses and physicians who helped with recruitment.

Author Contributions

SZ developed the idea for the manuscript. JM wrote the first draft, with edits made by SZ, JE, MM, CT, RSA and PV. BMB, AC, LE, SH, LL, TLON, AC, and BR contributed to the analysis meetings and provided comments on drafts.

Competing Interests

For peer review only

All authors have completed the ICMJE uniform disclosure form at www.icmje.org/coi_disclosure.pdf and declare: no support from any organisation for the submitted work; no financial relationships with any organisations that might have an interest in the submitted work in the previous three years; no other relationships or activities that could appear to have influenced the submitted work.

Copyright Statement

The Corresponding Author has the right to grant on behalf of all authors and does grant on behalf of all authors, a worldwide licence to the Publishers and its licensees in perpetuity, in all forms, formats and media (whether known now or created in the future), to i) publish, reproduce, distribute, display and store the Contribution, ii) translate the Contribution into other languages, create adaptations, reprints, include within collections and create summaries, extracts and/or, abstracts of the Contribution, iii) create any other derivative work(s) based on the Contribution, iv) to exploit all subsidiary rights in the Contribution, v) the inclusion of electronic links from the Contribution to third party material where-ever it may be located; and, vi) licence any third party to do any or all of the above.

Ethics

Approvals for research ethics and governance were obtained separately according to the requirements in each country as follows:

England: Research Ethics Service reference 14/NS/1035

Denmark: The Biomedical Research Ethics Committee System Act does not apply to this project, as the project does not implicate the use of human biological materials. Standard ethical protocol according to the American Anthropological Association was followed.

Sweden: Regional Ethics Board, Lund, Sweden, reg. no 2014/819

For peer review only

Informed consent

All participants signed a consent form before taking part in an interview. Participants in England signed a second form after interview giving permission for their interview to be available to researchers at the University of Oxford, and other universities under licence from Oxford, for use in research, teaching, and other publications.

Funding statement and independence of the researchers

This paper presents independent research funded by organisations from 3 European countries as follows:

In the UK, the study was supported by the National Awareness and Early Diagnosis Initiative (NAEDI), http://www.naedi.org.uk. The contributing partners include: Cancer Research UK; Department of Health, England; Economic and Social Research Council; Health and Social Care Research and Development Division, Pubic Health Agency, Northern Ireland; National Institute for Social Care and Health Research, Wales; and the Scottish Government. This funding also covered the costs associated with the comparative analysis meetings in Denmark and Sweden and funded translation of the Danish and Swedish material for publications.

In Denmark, the study was supported by the Research Centre for Cancer Diagnosis in Primary Care funded by The Danish Cancer Society and the Novo Nordic Foundation.

In Sweden, the study was supported by the Vårdal Foundation; the Strategic Research Program in Care Sciences (SFO-V), Umeå University; the Cancer Research Foundation in Northern Sweden; and from government funding of clinical research within the National Health Service, Sweden.

For peer review only - http://bmjopen.bmj.com/site/about/guidelines.xhtml

The views expressed in this paper are those of the authors, and not necessarily those of the NAEDI, Danish and Swedish funding partners.

Role of the sponsor

The study sponsor(s) or funder(s), had no role in the study design; the collection, analysis, and interpretation of data; writing of the report; or in the decision to submit the article for publication.

Access of researchers to all the data

All researchers in each country had access to all the data gathered in their own country. Interview transcripts were not translated and were not shared between countries, but Swedish and Danish interview extracts were translated by the bi-lingual researchers for the analytic discussions. The team lead in each country (SZ, RSA and BR) takes responsibility for the integrity of the data and the accuracy of the data analysis.

Transparency declaration

The lead author (the manuscript's guarantor) affirms that the manuscript is an honest, accurate, and transparent account of the study being reported; that no important aspects of the study have been omitted; and that any discrepancies from the study as planned (and, if relevant, registered) have been explained.

Data sharing statement

In Denmark the data are available for secondary analysis in conjunction with members of the research group named Comparative Cancer Experiences.

For peer review only - http://bmjopen.bmj.com/site/about/guidelines.xhtml

In England the participants gave informed consent for data to be copyrighted to the University of Oxford for secondary analysis, broadcasting, publication and teaching.

In Sweden the data are available for secondary analysis in conjunction with members of the research group named Comparative Cancer Experiences.

For peer review only

References

1. Forbes LJ, Simon AE, Warburton F, et al. Differences in cancer awareness and beliefs between Australia, Canada, Denmark, Norway, Sweden and the UK (the International Cancer Benchmarking Partnership): do they contribute to differences in cancer survival? *British journal of cancer* 2013;108(2):292-300. doi: 10.1038/bjc.2012.542
2. Rose PW, Rubin G, Perera-Salazar R, et al. Explaining variation in cancer survival between 11 jurisdictions in the International Cancer Benchmarking Partnership: a primary care vignette survey. *BMJ Open* 2015;5(5):e007212. doi: 10.1136/bmjopen-2014-007212
3. Friedemann Smith C, Whitaker KL, Winstanley K, et al. Smokers are less likely than non-smokers to seek help for a lung cancer 'alarm' symptom. *Thorax* 2016;71:659-61.
4. Sele LM, Elnegaard S, Balasubramaniam K, et al. Lifestyle factors and contact to general practice with respiratory alarm symptoms-a population-based study. *BMC Fam Pract* 2016;17:47. doi: 10.1186/s12875-016-0444-9
5. Chapple A, Ziebland S, McPherson A. Stigma, shame, and blame experienced by patients with lung cancer: qualitative study. *Bmj* 2004;328(7454):1470. doi: 10.1136/bmj.38111.639734.7C
6. Coleman MP, Forman D, Bryant H, et al. Cancer survival in Australia, Canada, Denmark, Norway, Sweden, and the UK, 1995–2007 (the International Cancer Benchmarking Partnership): an analysis of population-based cancer registry data. *The Lancet* 2011;377(9760):127-38. doi: 10.1016/s0140-6736(10)62231-3
7. De Angelis R, Sant M, Coleman MP, et al. Cancer survival in Europe 1999–2007 by country and age: results of EUROCARE-5—a population-based study. *The Lancet Oncology* 2014;15(1):23-34. doi: 10.1016/s1470-2045(13)70546-1
8. Richards MA. The size of the prize for earlier diagnosis of cancer in England. *British journal of cancer* 2009;101 Suppl 2:S125-9. doi: 10.1038/sj.bjc.6605402
9. Abdel-Rahman M, Stockton D, Rachet B, et al. What if cancer survival in Britain were the same as in Europe: how many deaths are avoidable? *British journal of cancer* 2009;101 Suppl 2:S115-24. doi: 10.1038/sj.bjc.6605401
10. Hiom SC. Diagnosing cancer earlier: reviewing the evidence for improving cancer survival. *British journal of cancer* 2015;112 Suppl 1:S1-5. doi: 10.1038/bjc.2015.23
11. Neal RD, Tharmanathan P, France B, et al. Is increased time to diagnosis and treatment in symptomatic cancer associated with poorer outcomes? Systematic review. *British journal of cancer* 2015;112 Suppl 1:S92-107. doi: 10.1038/bjc.2015.48
12. Chapple A, Ziebland S. Methodological and practical issues in cross-national qualitative research: Lessons from the literature and a comparative study of the experiences of people receiving a diagnosis of cancer. *Qualitative health research* 2017;[Online preview]
13. Butler J, Foot C, Bomb M, et al. The International Cancer Benchmarking Partnership: an international collaboration to inform cancer policy in Australia, Canada, Denmark, Norway, Sweden and the United Kingdom. *Health policy* 2013;112(1-2):148-55. doi: 10.1016/j.healthpol.2013.03.021
14. Rubin G, Berendsen A, Crawford SM, et al. The expanding role of primary care in cancer control. *The Lancet Oncology* 2015;16(12):1231-72. doi: 10.1016/s1470-2045(15)00205-3
15. DoH. Cancer Reform Strategy. London, England: Department of Health, 2007.
16. Simon AE, Waller J, Robb K, et al. Patient delay in presentation of possible cancer symptoms: the contribution of knowledge and attitudes in a population sample from

For peer review only

the United kingdom. *Cancer Epidemiol Biomarkers Prev* 2010;19(9):2272-7. doi: 10.1158/1055-9965.EPI-10-0219
17. Smith LK, Pope C, Botha JL. Patients' help-seeking experiences and delay in cancer presentation: a qualitative synthesis. *The Lancet* 2005;366(9488):825-31. doi: 10.1016/s0140-6736(05)67030-4
18. Whitaker KL, Scott SE, Winstanley K, et al. Attributions of cancer 'alarm' symptoms in a community sample. *PLoS One* 2014;9(12):e114028. doi: 10.1371/journal.pone.0114028
19. Olesen F, Hansen RP, Vedsted P. Delay in diagnosis: the experience in Denmark. *British journal of cancer* 2009;101 Suppl 2:S5-8. doi: 10.1038/sj.bjc.6605383
20. Greenhalgh T, Annandale E, Ashcroft R, et al. An open letter to The BMJ editors on qualitative research. *Bmj* 2016;352:i563. doi: 10.1136/bmj.i563
21. Pope C, Mays N. Qualitative Research: Reaching the parts other methods cannot reach: an introduction to qualitative methods in health and health services research. *Bmj* 1995;311(6996):42-45. doi: 10.1136/bmj.311.6996.42
22. Brown S, Castelli M, Hunter DJ, et al. How might healthcare systems influence speed of cancer diagnosis: a narrative review. *Social science & medicine* 2014;116:56-63. doi: 10.1016/j.socscimed.2014.06.030
23. OECD/EU. Health at a Glance: Europe 2016: State of Health in the EU Cycle. Paris: OECD Publishing 2016.
24. OECD. Consultations with doctors. Health at a Glance 2015: OECD Indicators. Paris: OECD Publishing 2015.
25. Wilkens J, Thulesius H, Schmidt I, et al. The 2015 National Cancer Program in Sweden: Introducing standardized care pathways in a decentralized system. *Health policy* 2016 doi: 10.1016/j.healthpol.2016.09.008
26. Vedsted P, Olesen F. A differentiated approach to referrals from general practice to support early cancer diagnosis - the Danish three-legged strategy. *British journal of cancer* 2015;112 Suppl 1:S65-9. doi: 10.1038/bjc.2015.44
27. Coyne IT. Sampling in qualitative research. Purposeful and theoretical sampling; merging or clear boundaries? *Journal of advanced nursing* 1997;26:623–30.
28. Guest G, Bunce A, Johnson L. How Many Interviews Are Enough?: An Experiment with Data Saturation and Variability. *Field Methods* 2006;18(1):59-82. doi: 10.1177/1525822x05279903
29. Levealahti H, Tishelman C, Ohlen J. Framing the onset of lung cancer biographically: narratives of continuity and disruption. *Psycho-oncology* 2007;16(5):466-73. doi: 10.1002/pon.1080
30. Andersen B, Cacioppo JT, Roberts DC. Delay in seeking a cancer diagnosis: delay stages and psychophysiological comparison processes. *Br J Soc Psychol* 1995;34:33-52.
31. Zola I. Pathways to the doctor: from person to patient. *Social science & medicine* 1973;7:677–89.
32. Andersen RS, Paarup B, Vedsted P, et al. 'Containment' as an analytical framework for understanding patient delay: a qualitative study of cancer patients' symptom interpretation processes. *Social science & medicine* 2010;71(2):378-85. doi: 10.1016/j.socscimed.2010.03.044
33. Evans J, Chapple A, Salisbury H, et al. It can't be very important because it comes and goes": patients' accounts of intermittent symptoms preceding a pancreatic cancer diagnosis: a qualitative study. *BMJ Open* 2014;4(e004215) doi: 10.1136/bmjopen-2013004215
34. Ziebland S, Chapple A, Evans J. Barriers to shared decisions in the most serious of cancers: a qualitative study of patients with pancreatic cancer treated in the UK.

For peer review only - http://bmjopen.bmj.com/site/about/guidelines.xhtml

Health expectations : an international journal of public participation in health care and health policy 2015;18(6):3302-12. doi: 10.1111/hex.12319

35. Pope C, Ziebland S, Mays N. Qualitative research in health care: Analysing qualitative data. *Bmj* 2000;320(7227):114-16. doi: 10.1136/bmj.320.7227.114
36. Wyke S, Adamson J, Dixon D, et al. Consultation and illness behaviour in response to symptoms: a comparison of models from different disciplinary frameworks and suggestions for future research directions. *Social science & medicine* 2013;86:79-87. doi: 10.1016/j.socscimed.2013.03.007
37. Tong A, Sainsbury P, Craig J. Consolidated criteria for reporting qualitative research (COREQ): a 32-item checklist for interviews and focus groups. *International Journal for Quality in Health Care* 2007;19(6):349–57. doi: 10.1093/intqhc/mzm042
38. Ziebland S, Coulter A, Calabrese J, et al. Understanding and Using Health Experiences: Improving patient care. Oxford: Oxford University Press 2013.
39. Renzi C, Whitaker KL, Winstanley K, et al. Unintended consequences of an 'all-clear' diagnosis for potential cancer symptoms: a nested qualitative interview study with primary care patients. *The British journal of general practice : the journal of the Royal College of General Practitioners* 2016;66(644):e158-70. doi: 10.3399/bjgp16X683845
40. Almond SC, Summerton N. Diagnosis in General Practice. Test of time. *Bmj* 2009;338:b1878. doi: 10.1136/bmj.b1878
41. Nicholson BD, Mant D, Bankhead C. Can safety-netting improve cancer detection in patients with vague symptoms? *Bmj* 2016:i5515. doi: 10.1136/bmj.i5515
42. Bankhead C, Heneghan C, Hewitson P, et al. Safety netting to improve early cancer diagnosis in primary care: development of consensus guidelines. Oxford: University of Oxford, 2011.
43. Ferlay J, Parkin DM, Steliarova-Foucher E. Estimates of cancer incidence and mortality in Europe in 2008. *Eur J Cancer* 2010;46(4):765-81. doi: 10.1016/j.ejca.2009.12.014
44. Andersen RA, Nichter M, Risør MB. Introduction: Sensations, Symptoms and Healthcare Seeking. *Anthropology in Action* 2017;24(1):1-5. doi: 10.3167/aia.2017.240101
45. McLachlan S, Mansell G, Sanders T, et al. Symptom perceptions and help-seeking behaviour prior to lung and colorectal cancer diagnoses: a qualitative study. *Family practice* 2015;32(5):568-77. doi: 10.1093/fampra/cmv048
46. Nicholson BD, Mant D, Neal RD, et al. International variation in adherence to referral guidelines for suspected cancer: a secondary analysis of survey data. *The British journal of general practice : the journal of the Royal College of General Practitioners* 2016 doi: 10.3399/bjgp16X683449
47. Power E, Wardle J. Change in public awareness of symptoms and perceived barriers to seeing a doctor following Be Clear on Cancer campaigns in England. *British journal of cancer* 2015;112 Suppl 1:S22-6. doi: 10.1038/bjc.2015.32
48. NICE. Suspected cancer: recognition and referral (NG12). NICE guideline: NICE, 2015.

For peer review only

COREQ Criteria Checklist

**Domain 1: Research team and reflexivity**<br><br>*Personal Characteristics*	Covered in article?	Page
1. Interviewer/facilitator Which author/s conducted the interview or focus group?	✓	8
2. Credentials What were the researcher's credentials? E.g. PhD, MD	✓	8, 1
3. Occupation What was their occupation at the time of the study?	✓	8
4. Gender Was the researcher male or female?	✓	8, 1
5. Experience and training: What experience or training did the researcher have?	✓	8, 1
Relationship with participants		
6. Relationship established: Was a relationship established prior to study commencement?	✓	9
7. Participant knowledge of the interviewer? What did the participants know about the researcher? e.g. personal goals, reasons for doing the research	✓	9
8. Interviewer characteristics What characteristics were reported about the interviewer/facilitator? e.g. Bias, assumptions, reasons and interests in the research topic.	✓	9
**Domain 2: study design**<br><br>*Theoretical framework*		
9. Methodological orientation and Theory What methodological orientation was stated to underpin the study? e.g. grounded theory, discourse analysis, ethnography, phenomenology, content analysis.	✓	9-10
Participant selection		
10. Sampling How were participants selected? e.g. purposive, convenience, consecutive, snowball	✓	8
11. Method of approach How were participants approached? e.g. face-to-face, telephone, mail, email	✓	8-9
12. Sample size How many participants were in the study?	✓	8
13. Non-participation How many people refused to participate or dropped out? Reasons?	No participants dropped out - our distributed recruitment methods means that we do not	

For peer review only

	know how many people saw information about the study	
Setting		
14. Setting of data collection Where was the data collected? e.g. home, clinic, workplace	✓	9
15. Presence of non-participants Was anyone else present besides the participants and researchers?	✓	9
16. Description of sample What are the important characteristics of the sample? e.g. demographic data, date	✓	8, 9
Data collection		
17. Interview guide Were questions, prompts, guides provided by the authors? Was it pilot tested?	✓	9
18. Repeat interviews Were repeat interviews carried out? If yes, how many?	NA	
19. Audio/visual recording Did the research use audio or visual recording to collect the data?	✓	9
20. Field notes Were field notes made during and/or after the interview or focus group?	Researchers kept their own field diaries with notes about the interviews – these were not intended for analysis	
21. Duration What was the duration of the interviews or focus group?	✓	9
22. Data saturation Was data saturation discussed?	✓	9
23. Transcripts returned Were transcripts returned to participants for comment and/or correction?	x	
**Domain 3: analysis and findings**<br><br>*Data analysis*		
24. Number of data coders How many data coders coded the data?	✓	9-10
25. Description of the coding tree Did authors provide a description of the coding tree?	n/a for this article	
26. Derivation of themes Were themes identified in advance or derived from the data?	✓	9-10
27. Software What software, if applicable, was used to manage the data?	✓	9
28. Participant checking Did participants provide feedback on the findings?	x (But PPI did)	10
Reporting		

For peer review only

29. Quotations presented Were participant quotations presented to illustrate the themes / findings? Was each quotation identified? e.g. participant number	✓	11-17
30. Data and findings consistent Was there consistency between the data presented and the findings?	✓	11-17
31. Clarity of major themes Were major themes clearly presented in the findings?	✓	11-17
32. Clarity of minor themes Is there a description of diverse cases or discussion of minor themes?	✓	11-17

For peer review only

BMJ Open

Patients' initial steps to cancer diagnosis in Denmark, England, and Sweden: what can a qualitative, cross country comparison of narrative interviews tell us about potentially modifiable factors?

Journal:	*BMJ Open*
Manuscript ID	bmjopen-2017-018210.R1
Article Type:	Research
Date Submitted by the Author:	11-Aug-2017
Complete List of Authors:	MacArtney, John; University of Oxford, Health Experiences Research Group, Nuffield Department of Primary Care Health Sciences<br>Malmström, Marlene; Lund University and Region Skåne, The Institute for Palliative Care; Lund University, Skane University Hospital, Department of Clinical Sciences Lund, Surgery<br>Overgaard Nielsen, Trine; Aarhus Universitet, Research Centre for Cancer Diagnosis in Primary Care, Research Unit for General Practice, Department of Public Health<br>Evans, Julie; University of Oxford, Health Experiences Research Group, Nuffield Department of Primary Care Health Sciences<br>Bernhardson, Britt-Marie; Karolinska Institutet, Department of Learning, Informatics, Management and Ethics<br>Hajdarevic, Senada; Umeå University, Department of Nursing<br>Chapple, Alison; University of Oxford, Health Experiences Research Group, Nuffield Department of Primary Care Health Sciences<br>Eriksson, Lars; Karolinska Institutet, Department of Learning, Informatics, Management and Ethics; City, University of London, School of Health Sciences<br>Locock, Louise; University of Oxford, Health Experiences Research Group, Nuffield Department of Primary Care Health Sciences<br>Rasmussen, Birgit; Lund University and Region Skåne, The Institute for Palliative Care; Lunds Universitet, Department of Health Sciences<br>Vedsted, Peter; Aarhus Universitet, Research Centre for Cancer Diagnosis in Primary Care, Research Unit for General Practice, Department of Public Health; Aarhus Universitet, Department of Clinical Medicine<br>Tishelman, Carol; Karolinska Institutet, Medical Management Centre, LIME; Karolinska Universitetssjukhuset, Center for Innovation<br>Andersen, Rikke; University of Aarhus, Research Centre for Cancer Diagnosis in Primary Care, Research Unit for General Practice, Department of Public Health<br>Ziebland, Sue; University of Oxford, Health Experiences Research Group, Nuffield Department of Primary Care Health Sciences
**Primary Subject Heading**:	Health services research
Secondary Subject Heading:	Qualitative research, General practice / Family practice
Keywords:	PRIMARY CARE, QUALITATIVE RESEARCH, International health services <

	HEALTH SERVICES ADMINISTRATION & MANAGEMENT

SCHOLARONE™
Manuscripts

For peer review only

Title: Patients' initial steps to cancer diagnosis in Denmark, England, and Sweden: what can a qualitative, cross country comparison of narrative interviews tell us about potentially modifiable factors?

List of authors

John I MacArtney[a], Senior Researcher.

Marlene Malmström[b, c], Senior Researcher.

Trine L Overgaard Nielsen[d], Research Assistant.

Julie Evans[a], Senior Researcher.

Britt-Marie Bernhardson[e], Senior Researcher.

Senada Hajdarevic[f], Senior Lecturer.

Alison Chapple [a], University Research Lecturer.

Lars E. Eriksson[e, g, h], Associate Professor.

Louise Locock[a], Associate Professor.

Birgit H Rasmussen[b, i], Professor.

Peter Vedsted[d, j], Professor.

Carol Tishelman[e, k], Professor.

Rikke Sand Andersen[d], Associate Professor.

Sue Ziebland[a], Professor.

[a] Health Experiences Research Group, Nuffield Department of Primary Care Health Sciences, University of Oxford, UK

[b]The Institute for Palliative Care, Lund University and Region Skåne, Sweden.

[c]Lund University, Skane University Hospital, Department of Clinical Sciences Lund, Surgery, Lund, Sweden

For peer review only

[d]Research Centre for Cancer Diagnosis in Primary Care, Research Unit of General Practice, Aarhus University, Denmark

[e]Department of Learning, Informatics, Management and Ethics, Karolinska Institutet, Sweden

[f]Department of Nursing, Umeå University, Sweden

[g]Department of Infectious Diseases, Karolinska University Hospital, Stockholm, Sweden

[h]School of Health Sciences, City, University of London, United Kingdom

[i]Lund University, Department of Health Sciences, Lund, Sweden

[j]University Clinic for Innovative Patient Pathways, Silkeborg Hospital, Department of Clinical Medicine, Aarhus University, Denmark.

[k]Center for Innovation, Karolinska University Hospital, Stockholm Sweden

Corresponding author:

John I MacArtney

Nuffield Department of Primary Care Health Sciences

University of Oxford

Radcliffe Observatory Quarter

Woodstock Road

Oxford

OX2 6GG

john.macartney@phc.ox.ac.uk

For peer review only

For peer review only - http://bmjopen.bmj.com/site/about/guidelines.xhtml

Abstract

Objectives

To illuminate patterns observed in International Cancer Benchmarking Programme studies by extending understanding of the various influences on presentation and referral with cancer symptoms.

Design

Cross-country comparison of Denmark, England, and Sweden with qualitative analysis of in-depth interview accounts of the pre-diagnostic process in lung or bowel cancer.

Participants

155 women and men, aged between 35 and 86 years old, diagnosed with lung or bowel cancer in the six months before interview.

Setting

Participants recruited through primary and secondary care, social media, and word-of-mouth. Interviews collected by social scientists or nurse researchers during 2015, mainly in participants' homes.

Results

Participants reported difficulties in interpreting diffuse bodily sensations and symptoms and deciding when to consult. There were examples of swift referrals by primary care professionals in all three countries. In all countries participants described difficulty deciding if and when to consult, highlighting concerns about access to GP appointments and overstretched primary care services, although this appears less prominent in the Swedish data.

For peer review only

It was not unusual for there to be more than one consultation before referral and we noted two distinct patterns of repeated consultation: (1) Situations where the participant left the primary care consultation with a plan of action about what should happen next. (2) Participants were unclear about under which conditions to return to the doctors. This second pattern sometimes extended over many weeks during which patients described uncertainty, and sometimes frustration, about if and when they should return and whether there were any other feasible investigations. The latter pattern appeared more evident in the interviews in England and Denmark than Sweden.

Conclusion

We suggest that if clear action plans, as part of safety netting, were routinely used in primary care consultations then uncertainty, false reassurance, and the inefficiency and distress of multiple consultations could be reduced.

For peer review only - http://bmjopen.bmj.com/site/about/guidelines.xhtml

Strengths and limitations of this study:

- This study provides a social science informed, qualitative cross-country comparison of 155 in-depth interviews with bowel and lung cancer patients recruited within six months of a diagnosis in Sweden, Denmark and England.
- The methods we use provide insight into why and how potentially modifiable factors identified by the International Cancer Benchmarking Project - including response to symptom experiences, differences in willingness to consult, how people negotiate access to healthcare, and what happens during consultations - affect the time to diagnosis in a primary care setting.
- Our analysis was able to show how patients' narratives suggested differences between the three countries in the clarity and communication of action plans at the end of primary care consultations.
- The study was limited to patient interviews and no 'first hand' observational data were available to compare accounts.

For peer review only

Introduction

Background

No single factor is likely to explain why differences in cancer outcomes persist between high-income countries with universal health coverage. The International Cancer Benchmarking Partnership (ICBP), which compares cancer registry data and cross country surveys, has explored several hypotheses about why variations occur. These studies have shown a number of potentially modifiable factors, for example, that patterns in public knowledge about cancer awareness and beliefs were not clearly associated with variations in survival across countries.[1] The ICBP studies have also drawn attention to different patterns in public willingness to consult a Primary Care professional (PCP), with patients in the UK reporting particular concern 'not to waste the doctor's time'.[1] There are also cross country differences in general practitioners (GPs) expressed willingness to refer a patient with suspicious symptoms at their first presentation and PCPs have reported that investigations such as computerised tomography (CT) and Magnetic Resonance Imaging (MRI) were harder to access and results took longer in England and Denmark, than in Sweden.[2] There seems to be a relationship between smoking behaviour and willingness to consult: in separate, recent, studies both English [3] and Danish smokers [4] have been shown to be less likely than non-smokers to consult with red flag lung cancer symptoms. It has been suggested that this is related to the shame, blame and stigma associated with lung cancer as a smoking related disease.[5] Socio-economic deprivation, inequalities in service provision and regional differences in expectations about the likely consequences of seeking care further contribute to a complicated picture.

Denmark, England, and Sweden (all ICBP participants) were selected for this comparison because survival for lung and bowel cancer between 1995 and 2007 were persistently higher

For peer review only - http://bmjopen.bmj.com/site/about/guidelines.xhtml

in Sweden than in Denmark or England, particularly in the first year after diagnosis.[6] We chose lung and bowel cancer because they affect both genders, are the two most common causes of death from cancer across Europe,[7] their symptomatology is often diffuse or vague, and these cancers are often prone to late stage diagnosis. Earlier diagnosis has become a key healthcare research and policy target in all three countries.[8 9 10] Analyses of potentially modifiable factors have drawn attention to between country differences in stage at diagnosis and access to good care, diagnostics and screening.[11] Late presentation and long diagnostic intervals are also potentially modifiable contributory factors to poor survival.[6 11-15] Cross country comparative research can suggest routes for service redesign as well as hypotheses for further research.[16] Social and health sciences have suggested potentially modifiable factors affecting the time to diagnosis include public response to symptom experiences,[1 3-5 17-26] differences in willingness to consult, [1 3-5 17-24] how people negotiate access to healthcare,[1 18 22 27 28] what happens during consultations,[2 27 29-31] and access to diagnostic tests.[2] While surveys and cancer registry data can provide excellent high level comparative data showing patterns of association, in-depth qualitative research is needed to help illuminate why and how observed variations may occur.[32 33] We therefore designed a study to contribute a qualitative cross-country comparative analysis of narrative interviews with patients recently diagnosed with lung or bowel cancer in Denmark, England, and Sweden. In this paper the research question we ask is, what might explain some of the variations identified in the ICBP?

The healthcare systems in Denmark, England and Sweden.

All three countries have primarily tax-based health systems. However, in Sweden patient co-payments vary from no payment to 300SEK per primary care visit, depending on county council, up to a total of ~1,100SEK (£100) per year.[34] No co-payments are made in England

For peer review only - http://bmjopen.bmj.com/site/about/guidelines.xhtml

and Denmark, although there are prescription charges in all three countries. In Sweden two-thirds of GPs are publically employed, whereas in Denmark and England most are self-employed.[34] Sweden spends over 20% more on healthcare per head of population than England.[35] Contact with primary care varies between the three countries: Swedish patients consult PCPs least frequently (2.9 per annum) followed by Denmark (4.7 per annum) and UK (5.0 per annum, which includes telephone consultations).[36] In 2015 England withdrew guidance stipulating access for urgent appointments within 48 hours.[34] In some settings in Sweden, a nurse is the first person a patient speaks to when seeking an appointment.[34] Most first contact in Denmark and England is with a receptionist, who may pass the patient to a nurse to be triaged. In Denmark some receptionists have been trained to recognise potentially serious symptoms.[34] GPs have a central role in making referrals to lung and colorectal specialists in all three countries [34] The national cancer plan in Sweden is regionally administered and focused on secondary care[37], whereas in England and Denmark specific clinical guidance and targets for PCPs are also included.[34] England (implemented 2006) and Denmark (implemented 2014) have national cancer screening programmes for bowel cancer. In Sweden bowel screening was implemented regionally, with some regions having some form of programme from 2008. Currently it is recommended for 60-74 year olds, although this again varies by region. Denmark has increased rapid access clinics and access to investigations, while in England referral management systems have been interspersed between the GP and secondary care.[34 38]

Methods

Sampling and recruitment

We interviewed 155 people during 2015 who were within six months of a diagnosis of lung or bowel cancer in the three countries. Purposive sampling[39] within each country was used to

For peer review only - http://bmjopen.bmj.com/site/about/guidelines.xhtml

achieve gender balance and variation across age, urban and rural locations, and pathway to diagnosis (see Table 1). An experienced sociologist in England (JM) and an anthropologist in Denmark (TON) recruited and conducted all the interviews in their countries, while three experienced nurse researchers collaborated in different regions of Sweden (BMB, SH, and MM). Participants were initially approached by treating clinicians in hospital clinics in all three countries. To reach data saturation, in England and Denmark this approach was supplemented with some additional recruitment from support groups, social media, and word-of-mouth. All potential participants were provided with an information sheet that included the rationale for the study and an explanation of what would be involved if they consented to take part in the study. Data saturation[40] was judged to have been reached in the analytic categories for all three countries before recruitment closed.

		Denmark		England		Sweden	
		BC	LC	BC	LC	BC	LC
**Number of participants**		28	22	25	20	30	30
**Percentage female**		46%	36%	48%	50%	47%	50%
**Age range**	**31-50**	2	0	4	*2*	2	2
	**51-70**	19	15	13	12	14	21
	**71-90**	7	7	8	6	14	7

Table 1: Participant demographic characteristics across the three countries

Data collection

Interviews took place in participants' homes, unless they preferred another location. One-to-one interviews were preferred, but a small number of participants requested a non-

For peer review only - http://bmjopen.bmj.com/site/about/guidelines.xhtml

participating family member to be present. Interviews started with an open ended question: "Could you start by telling me, in your own words and in as much detail as you want, about everything that has happened since you first started to suspect there might be a problem with your health?" Interviews lasted between 45 minutes to 1.5 hours. During the interview the researchers used a semi-structured topic guide based on social science theories, and the cancer research literature (highlighted in the Introduction), including factors related to the diagnostic interval.[1-5 17-31] The research team had extensive discussions about the topics to ensure comparable data were collected.[41] Interviews were audio recorded and transcribed verbatim.

Analysis

Monthly teleconferences with the field research team (all of whom had a high level of spoken and written English) were held throughout the development of the topic guide, recruitment, data collection and analysis phases. Interview accounts were analysed for narrative themes that structured participant experiences. To do this, in each country interview transcripts were imported into specialist computer software (NVivo 10) for organising textual data and were coded by the interviewing researchers. The three research teams conducted separate thematic analysis with their own data using a coding frame developed through discussion in the teleconferences and based on the (anticipated) themes from the topic guide and on emergent themes.[42] One two-day and one four-day analysis and writing workshops were held with the research teams from all three countries. Anticipated issues drew on existing knowledge and theoretical insights.[1-5 17-31] Emerging analyses were iteratively tested within and between datasets. Allocations between the analysis categories (including identifying participants who described leaving the PCP consultation with an understanding of what should happen next and those who reported uncertainty about what to do) was discussed among the field

For peer review only

researchers. Direct quotes from the interviews (translated into English by the bi-lingual researchers) are used to illustrate the results. Analysis and findings are presented in accordance with relevant COREQ[43] criteria for reporting qualitative data and are consistent with interpretive approaches to analysis.[44]

Patient involvement

Public and Patient Involvement (PPI) was conducted in accordance with good practice in each country. Representatives in England with experience of bowel or lung cancer were involved in preparing the funding application, the study design and the multidisciplinary advisory group, as well as advising on recruitment strategies, including the patient information sheets. In all three countries PPI members were invited to comment on the draft interview topic guide. A group of three English PPI members met with the research team to look at a subset of the English data. Drafts of study papers have been circulated to the advisory panel. Summaries of the main study findings, prepared for a stakeholder final event in 2017, will be circulated to all study participants who expressed interest in seeing the results.

Results

We compared the three sets of interviews to consider the following potentially modifiable steps, drawn from the literature, on the pre-diagnosis pathway: people's initial responses to bodily sensations and the transformation of these into symptoms justifying medical advice; accounts of accessing medical help; what happened in the consultation; and, planning for follow-up.

For peer review only

Awareness and responses to signs and symptoms

The first potentially modifiable factors are the type of sensations and bodily changes people considered to justify seeking help. Participants in all three countries described considerable uncertainty about the possible implications of bodily sensations and when or if they should be considered symptoms warranting medical advice, especially if they felt well. For example, apart from some blood in his stools a participant in England said he had been "fit as anything and not feeling ill at all" (England-BC18). Even those who looked up their symptoms and risk factors online were reassured if they found that relatively few items on the checklists applied to them. People reasoned that they must be low risk if they felt they had a good diet, were physically active, non-smokers (or long term ex-smokers), generally fit, relatively young, or without a family history of cancer. This reasoning suggested that some patients expected there to be a linear relationship between healthy input and outcomes. For example, a participant in Denmark who acknowledged "[I] smoke and [drink] alcohol and [eat] too much meat…" went on to suggest that other behaviours offered some protection from the profile of a typical cancer patient:

> Denmark-BC01: I have read about this, I'm very much into organic food. All the things you say can cause cancer… you don't know […]. I have at least lived a healthy life with vegetables everyday…and fruit, so maybe I'm not the typical cancer patient.

Persistent coughs, pains, stomach aches, changes in bowel habits, bleeding or constipation were attributed to everyday causes or to more familiar diseases such as influenza, pneumonia, Irritable Bowel Syndrome (IBS) or hemorrhoids.[21] People with lung cancer, who smoked cigarettes, reasoned that a persistent cough was a 'normal' bodily response to smoking. Thus, even those who are aware that they have risk factors, or suspicious symptoms, may conclude that overall their risk is low and not seek medical help. Consistent with Zola's classic work

For peer review only - http://bmjopen.bmj.com/site/about/guidelines.xhtml

on triggers to the consultation, when people *did* consult this was usually because the symptoms: interfered with daily life; became evident to others who encouraged them to consult; or, exceeded a self-imposed time limit[22], rather than because they suspected that they might have cancer.

Access to primary care

The second potentially modifiable factor is whether the participant felt able to access primary care. There were some differences apparent in accounts from the three countries. In England, people talked about long waiting times for 'non-urgent' appointments or (echoing ICBP findings) said they were reluctant to trouble busy GPs with potentially minor illnesses that might be treatable with over-the counter remedies.[1] A participant in England, later diagnosed with bowel cancer, had concluded, "It's haemorrhoids, leave it. Doctors are busy with other things" (England-BC20). Another participant in England with lung cancer explained why she hadn't consulted with a cough:

> England-LC12: When you hear that the GPs are so busy and being bothered by, you know, minor illnesses it makes you reluctant to go with something that you think [is minor] . . . I was, well, "What can the doctor do? They won't want to give you antibiotics for a virus." That was what I, I thought. And I just didn't want to really bother the doctor.

A participant in Denmark similarly reflected upon an implication of GPs' time pressures:

> Denmark-LC15: The system is not always that easy. First you have to convince the secretary, that you need an appointment, right. That is what happens when they are too busy.

For peer review only - http://bmjopen.bmj.com/site/about/guidelines.xhtml

In contrast, we were struck that accounts in Sweden rarely included detailed justifications for using the doctor's time, nor did they suggest that the onus was on the participant to determine whether the matter was serious, or urgent. For example, a participant in Sweden diagnosed with bowel cancer said:

> Sweden-BC051: It began like this, that I found a little blood ... I can say in the stool, very little . . . And I tend to ignore such things ... but this was just strange enough, so I thought, "no, I've got to go to the health centre and hear what it is". And so I did.

Another participant in Sweden with lung cancer said, "It's better to go one time too many than one too few" (Sweden-LC102). This sentiment was not expressed in any of our interviews in England.

During the consultation

The third potentially modifiable factor is what happens when the patient presents with symptoms. Seeking healthcare is not just a matter of making an appointment, but also of being able to present one's problem and be heard.[9] In all three countries there were examples of a single consultation and a prompt referral. A participant in England said,

> England-BC18: Our local GP was brilliant, she said, "I'm not gonna do the kind of normal give you this stool sample packet, all that kinda stuff. I'm gonna hotline you straight to [hospital] down the road there for an endoscopy. Skip all the intermediate stuff and go straight into endoscopy", which was a fabulous decision, if you think of it.

Similarly a participant in Denmark explained,

For peer review only - http://bmjopen.bmj.com/site/about/guidelines.xhtml

> Denmark-BC13: The stomach pains had been going on for a while, but one day I just could not stand it anymore and drove straight down to my doctor's office. My doctor examined it and immediately said: "I will send you off to the hospital". And I was [sent off] in the afternoon. That was quick.

A participant in Sweden with bowel cancer recalled the GP saying, "No, I cannot see anything, but for safety's sake I will send you to a specialist […] so they get a closer look at it" (Sweden-BC051).

There were also examples of participants in all three countries who told us that they had returned to the doctors over periods of weeks or even months, before they were referred for further investigations. Some participants in England and Denmark diagnosed with lung cancer said they had been repeatedly treated with antibiotics for chest infections. Participants diagnosed with bowel cancer from all three countries described inconclusive consultations for presumed constipation, haemorrhoids, IBS, Crohn's, or psychological causes; however in our data this appeared more often in interviews from Denmark and England. This example from England typifies this:

> England-BC09: . . . each time it was treated obviously as constipation. There was never even any mention of anything else. . . He's (the GP) a very, "I can do it. I can fix it. I can do it". But each time I come away, it was so sort of disheartening, because it seemed to be about what he could do and not actually helping the situation. But I did have, I had all sorts of laxatives from him. It's as if like we just keep trying to do this, but I was in agony each time I took it. I did explain that each time. . . . But, yeah, nothing really. It was more like the repetitive sort of appointment.

For peer review only

Making plans for follow-up

The fourth potentially modifiable factor is what happens after those consultations which do not include a referral. We looked in detail at the accounts of those participants who reported two or more presentations before referral and noted two different types of experience, depending on whether or not the patient reported leaving the consultation knowing what to do next.

<u>Repeated consultation(s) with patient awareness of next steps</u>

First, there were participants in all three countries who described leaving the consultation with an understanding about what to expect, including how long to wait for symptoms to abate, what should prompt them to return, and what would happen after that. For example this participant in Sweden said,

> Sweden-LC129: And then the [GP] said, "No it's nothing. Your lungs sound very healthy, but you've had this cough for the last eight weeks and if it does not disappear in another week, then please call us. Then we'll see."

The participants in this 'planned' group had also known what would be likely to happen if and when they did return; all three countries included examples of people who knew that further treatments or investigations would be needed if the symptoms did not resolve:

> England-LC17: And he [GP] prescribed two weeks antibiotics. And then he said that after that, if it didn't make any difference then he might prescribe omeprazole and maybe then a chest x-ray.

Follow-up advice can be reinforced by practice staff when delivering a normal test result in the course of their diagnostic investigations.[45] For example a participant in Sweden reported

For peer review only - http://bmjopen.bmj.com/site/about/guidelines.xhtml

that the practice nurse said, "if [it] gets worse – for example if you lose weight – contact us directly" (Sweden-BC055).

Repeat consultation(s) with patient uncertainty about next steps

Second, there were participants who were unsure what would or *should* happen next, such as England-BC09 quoted above who described a "disheartening" and "repetitive sort of appointment". These participants included those who worried that it was not appropriate to return to the doctor with the same symptoms and those who had been reassured at the initial consultation. The following participant in Denmark had requested a colonoscopy after many months of unusual symptoms and several investigations for other possible causes.

> Denmark-BC05: I can see now that I had these symptoms for three years. They [the doctors] thought that it was only the myalgia. But it probably was the colon cancer, even though I did not have the usual symptoms, you know. No bleeding, or diarrhoea or constipation. Just this strange heaviness in my lower parts. So I had an ultrasound of my bladder and kidneys, an x-ray of my lumbar area… And then I finally asked for a colonscopy. My doctor asked me if I was sure… of course I was… but I had not thought of cancer.

In these accounts people had been unsure what to expect and left without a plan about what to do next. Unsurprisingly, people who had presented on two, three or more occasions described some frustration. In retrospect, some wondered if the PCP had decided that a "test of time" or "wait-and-see" was the best course of action, without sharing their reasoning.[30 31]

> England-BC03: And the simple thing and I agree the most obvious thing was, you know, piles and irritable bowel syndrome . . . I guess they, the thought was, "Well

For peer review only - http://bmjopen.bmj.com/site/about/guidelines.xhtml

> we'll send her away with that for now and if doesn't work, she's bound to come back."

Accounts of repeated consultations, without an action plan communicated to the patient, featured more in the interviews with patients in England and Denmark than in Sweden. Patients may welcome reassurance from their PCP about their suspicious symptoms, and this may not be a problem if the practitioner takes responsibility for following up the patient as part of safety netting – the process of communicating with patient what to expect, documenting any action plans, and following the patient up as agreed.[46] A Swedish lung cancer patient explained that the GP had telephoned to see 'how she was' after a gastroscopy and made an urgent referral:

> Sweden-LC123 I got a referral to ultrasound of my thyroid and upper gastro. And then they discovered in a gastroscopy that I had oesophagitis . . . [a week later the GP] called to ask how I was. I said, "now it is bad", I said. "I think I have to go to the hospital urgently as it is now, it's ... I cannot breathe". "Come here", she said, "and we'll make an urgent referral".

Discussion

Principal findings

Our international qualitative comparison has considered four potentially modifiable features of the pre-diagnostic experience of lung and bowel cancer patients. Across the countries there are many similarities in how symptoms were experienced and in awareness of what the symptoms might mean. Our analysis suggests differences between countries in people's accounts of their willingness to consult and re-consult, their descriptions of what happened during consultations and, for those who did not receive an immediate referral, their confusion or clarity about what they should look for, when to return and what to expect. Reluctance and

For peer review only - http://bmjopen.bmj.com/site/about/guidelines.xhtml

practical barriers to consulting the GP, and uncertainty about whether and when to re-attend after an initial consultation, appeared more evident in interviews in Denmark and England. This indicates the benefit of communication of clear action plans to patients, including information on symptom development, and other safety netting approaches,[47] which could well be developed more. PCPs action plans might include a 'test of time', yet this reasoning should be clearly communicated to the patient. This could also mean that patients would receive more timely referrals, and avoid the repeated visits to primary care which featured particularly in English and Danish interviews.[31]

Strengths and weaknesses of the study

The study design – a cross-cultural comparison using in-depth qualitative interview – is the first of its kind to study the route to cancer diagnosis. Our findings suggest potentially modifiable factors that could improve timely diagnosis of cancer in all three countries, although the scope for improvement may be greater in England and Denmark.

This qualitative study was designed to illuminate findings from the ICBP studies and is intended to be indicative rather than conclusive. Findings extend observations from other research that highlights the centrality of the primary care encounter in pre-diagnostic experiences. Participants' cancer disease stage is likely to have affected their experiences. To achieve diversity we recruited people who were diagnosed with very early stage and others diagnosed with advanced disease in all three countries. Participants who were recruited via social media in Denmark and England may also be thought to have had different experiences, than those recruited via clinics. All participants were, or had been, under specialist cancer care and within six months of diagnosis. Each data set was analysed by the country specific

For peer review only - http://bmjopen.bmj.com/site/about/guidelines.xhtml

team, thus benefitting from familiarity with healthcare context, language and culture. These country specific analyses were shared as part of a careful collaborative process, involving workshops, teleconferences and several drafts and further targeted analyses. Along with the teleconferences, these meetings helped to mitigate any potential for differences in the data arising from differences in country or disciplinary approaches to interviewing or analysis.[41]

Strengths and weaknesses in relation to other studies

This study was designed to explore whether a cross-country comparison of qualitative interviews could help to illuminate findings from the ICBP survival analyses and surveys. While the high level comparisons of large quantitative data bases and surveys provide invaluable information, qualitative studies can suggest why these patterns may occur and identify avenues for service redesign, such as action plans, some of which could be implemented without further research. We did not observe the consultations described by the patients and it is possible that the PCPs involved did try to communicate an action plan, even if it did not feature in the patient's account. Again, we have no reason to suspect that variations in recall would differ between the countries. It is well known that public knowledge of cancer symptoms is insufficient to prompt people to seek help, as recently demonstrated in ICBP surveys.[1] Our work was guided by insights from the social and healthcare sciences into the complexity inherent in decisions to consult. These are influenced by people's ideas about their own candidacy, related to family history and health behaviours, as well as their access to care.[25 28 48] Evans et al have shown that patients, and their doctors, may be falsely reassured if a symptom comes and goes, reasoning that intermittent symptoms are unlikely to be serious.[24] Renzi and colleagues have drawn attention to the potential negative consequences of reassurance through an 'all-clear' result from investigations, which could be given with or without an encouragement to return.[45]

For peer review only

This study provides further insight into ICBP findings of how cancer symptoms are managed in primary care, in particular how delays may be compounded when a patient is not referred promptly and no clear plan is communicated about the circumstances in which they should return for further consultation or investigations.[2 49] Current research is exploring how and why planning, communicating action plans to patients, and following-up are operationalised as part of safety netting in English primary care and what approaches are acceptable to both GPs and patients.[46]

Implications for clinicians and policy makers

There are several important differences between these three countries, for example in access to secondary care, in spending on health services and in the numbers of licensed practitioners. The health system in England has less money, fewer doctors and nurses and fewer hospital beds then Sweden or Denmark.[34-36] These factors may yet prove critical in limiting improvements in cancer survival. Patients in Sweden consult their GP less often than patients in England or Denmark, but for longer.[36] Shorter consultations may not be efficient – patients in England consult their GPs for nearly one hour per year, yet spread over five appointments while, on average, patients in Sweden visit primary care twice a year, but for a mean of 20 minutes per appointment.

Through our international comparison we have been able to identify opportunities for pathway and service improvement that could promote earlier stage diagnosis. The perception of systemic barriers (e.g. access to PCP appointments) may prevent patients seeking timely help, even in the presence of potentially alarming symptoms (e.g. blood in stools or a cough that lasts for three weeks). While awareness of symptoms may be important, there is a limit to

For peer review only - http://bmjopen.bmj.com/site/about/guidelines.xhtml

how much effect increasing public awareness could have in settings where access is limited and people are uncertain when to consult, or re-consult.[23 26]

The importance of safety netting was emphasised in the 2015 guidance for suspected cancer from the UK National Institute for Health and Care Excellence[50] and a 2011 Delphi study of British GPs and primary care researchers led to publication of suggestions for how to make safety netting more effective.[46] In Denmark, direct access to investigation from general practice, with the aim to lower the GPs' threshold for referring and lengthy 'wait-and-see' approaches[30], is being investigated along with a focus on safety-netting. Our findings suggest that clearly communicated action plans at the end of *any* primary care consultation could be encouraged, without the need for further research. This would be likely to improve the patient's experience and may also reduce demand for repeated appointments and diagnostic delay.

Unanswered questions/need for further research

Research is needed into ways of redesigning primary care services in all three countries to reduce (perceived) barriers to consultation. NICE has recommended that British PCPs employ safety-netting to reduce delays in the diagnosis of cancer, but there has been little research to understand what constitutes best practice. Further research might address how a "test of time" or "wait-and-see" approach that includes clear planning affects the number of follow-up visits and cancer stage at diagnosis. We do not know how action planning is communicated in the consultation, nor what effect this may have on the length or frequency of consultations. This could be explored in future mixed methods research, ideally including conversation analysis of the consultation. Cross country differences in referral and follow up practices in lung cancer and bowel cancer patients could be investigated further, especially in

For peer review only - http://bmjopen.bmj.com/site/about/guidelines.xhtml

the light of recent studies suggesting that smokers (in Denmark and England) may be less likely to consult. Is the same pattern apparent in Sweden – and if not, why not?

Conclusion

If all primary care consultations concluded with a clear description of what should happen next and the circumstances in which the patient should return, this might avert some of the uncertainty and repeated consultations that were described in these interviews. We conclude that there is an opportunity for clearer communication and better follow-up strategies in primary care – neither of which will put additional pressure on specialist services, yet hold the potential to reduce diagnostic delays with few additional resources.

For peer review only

Other BMJ requirements

Acknowledgements

We are extremely grateful to the people who took part in this research in all three countries, and to the study advisory panel, including patient and public representatives, who helped design the study and provided comments on an earlier draft of this manuscript. We also acknowledge the support of the National Institute for Health Research, through the Clinical Research Network, who helped recruit patients into the English arm of the study. We would also like to thank all those who helped to recruit participants: In England we would like to thank the NHS Hospital Trusts that assisted with this study and Patients Active in Research: Thames Valley, along with the following charities who posted links or circulated our details on social media: Beating Bowel Cancer, Bowel Cancer UK, Roy Castle Lung Cancer Foundation, British Lung Foundation, and healthtalk.org. In Denmark we want to thank the Lung Cancer Patient organisation, as well as the Colon Cancer Patient organisation for assisting us in recruiting patients. We also wish to thank the local support groups of the national Danish Cancer Society, as well as the local oncology departments in various regions of Denmark. In Sweden we would like to thank the nurses and physicians who helped with recruitment.

Author Contributions

SZ developed the idea for the manuscript. JM wrote the first draft, with edits made by SZ, JE, MM, CT, RSA and PV. BMB, AC, LE, SH, LL, TLON, AC, and BR contributed to the analysis meetings and provided comments on drafts.

Competing Interests

For peer review only

All authors have completed the ICMJE uniform disclosure form at www.icmje.org/coi_disclosure.pdf and declare: no support from any organisation for the submitted work; no financial relationships with any organisations that might have an interest in the submitted work in the previous three years; no other relationships or activities that could appear to have influenced the submitted work.

Copyright Statement

The Corresponding Author has the right to grant on behalf of all authors and does grant on behalf of all authors, a worldwide licence to the Publishers and its licensees in perpetuity, in all forms, formats and media (whether known now or created in the future), to i) publish, reproduce, distribute, display and store the Contribution, ii) translate the Contribution into other languages, create adaptations, reprints, include within collections and create summaries, extracts and/or, abstracts of the Contribution, iii) create any other derivative work(s) based on the Contribution, iv) to exploit all subsidiary rights in the Contribution, v) the inclusion of electronic links from the Contribution to third party material where-ever it may be located; and, vi) licence any third party to do any or all of the above.

Ethics

Approvals for research ethics and governance were obtained separately according to the requirements in each country as follows:

England: Research Ethics Service reference 14/NS/1035

Denmark: The Biomedical Research Ethics Committee System Act does not apply to this project, as the project does not implicate the use of human biological materials. Standard ethical protocol according to the American Anthropological Association was followed.

Sweden: Regional Ethics Board, Lund, Sweden, reg. no 2014/819

For peer review only

Informed consent

All participants signed a consent form before taking part in an interview. Participants in England signed a second form after interview giving permission for their interview to be available to researchers at the University of Oxford, and other universities under licence from Oxford, for use in research, teaching, and other publications.

Funding statement and independence of the researchers

This paper presents independent research funded by organisations from 3 European countries as follows:

In the UK, the study was supported by the National Awareness and Early Diagnosis Initiative (NAEDI), http://www.naedi.org.uk. The contributing partners include: Cancer Research UK; Department of Health, England; Economic and Social Research Council; Health and Social Care Research and Development Division, Pubic Health Agency, Northern Ireland; National Institute for Social Care and Health Research, Wales; and the Scottish Government. This funding also covered the costs associated with the comparative analysis meetings in Denmark and Sweden and funded translation of the Danish and Swedish material for publications. During the study LL was Director of Applied Research at the Health Experiences Research Group, Nuffield Department of Primary Care Health Sciences, University of Oxford, and was supported by the National Institute for Health Research (NIHR) Oxford Biomedical Research Centre and the NIHR Collaboration for Leadership in Applied Health Research and Care Oxford (CLAHRC) at Oxford Health NHS Foundation Trust.

In Denmark, the study was supported by the Research Centre for Cancer Diagnosis in Primary Care funded by The Danish Cancer Society and the Novo Nordic Foundation.

For peer review only - http://bmjopen.bmj.com/site/about/guidelines.xhtml

In Sweden, the study was supported by the Vårdal Foundation; the Strategic Research Program in Care Sciences (SFO-V), Umeå University; the Cancer Research Foundation in Northern Sweden; and from government funding of clinical research within the National Health Service, Sweden.

The views expressed in this paper are those of the authors, and not necessarily those of the NAEDI, Danish and Swedish funding partners.

Role of the sponsor

The study sponsor(s) or funder(s), had no role in the study design; the collection, analysis, and interpretation of data; writing of the report; or in the decision to submit the article for publication.

Access of researchers to all the data

All researchers in each country had access to all the data gathered in their own country. Interview transcripts were not translated and were not shared between countries, but Swedish and Danish interview extracts were translated by the bi-lingual researchers for the analytic discussions. The team lead in each country (SZ, RSA and BR) takes responsibility for the integrity of the data and the accuracy of the data analysis.

Transparency declaration

The lead author (the manuscript's guarantor) affirms that the manuscript is an honest, accurate, and transparent account of the study being reported; that no important aspects of the

For peer review only

study have been omitted; and that any discrepancies from the study as planned (and, if relevant, registered) have been explained.

Data sharing statement

In Denmark the data are available for secondary analysis in conjunction with members of the research group named Comparative Cancer Experiences.

In England the participants gave informed consent for data to be copyrighted to the University of Oxford for secondary analysis, broadcasting, publication and teaching.

In Sweden the data are available for secondary analysis in conjunction with members of the research group named Comparative Cancer Experiences.

For peer review only

References

1. Forbes LJ, Simon AE, Warburton F, et al. Differences in cancer awareness and beliefs between Australia, Canada, Denmark, Norway, Sweden and the UK (the International Cancer Benchmarking Partnership): do they contribute to differences in cancer survival? *British journal of cancer* 2013;108(2):292-300. doi: 10.1038/bjc.2012.542
2. Rose PW, Rubin G, Perera-Salazar R, et al. Explaining variation in cancer survival between 11 jurisdictions in the International Cancer Benchmarking Partnership: a primary care vignette survey. *BMJ Open* 2015;5(5):e007212. doi: 10.1136/bmjopen-2014-007212
3. Friedemann Smith C, Whitaker KL, Winstanley K, et al. Smokers are less likely than non-smokers to seek help for a lung cancer ‘alarm’ symptom. *Thorax* 2016;71:659-61.
4. Sele LM, Elnegaard S, Balasubramaniam K, et al. Lifestyle factors and contact to general practice with respiratory alarm symptoms-a population-based study. *BMC Fam Pract* 2016;17:47. doi: 10.1186/s12875-016-0444-9
5. Chapple A, Ziebland S, McPherson A. Stigma, shame, and blame experienced by patients with lung cancer: qualitative study. *Bmj* 2004;328(7454):1470. doi: 10.1136/bmj.38111.639734.7C
6. Coleman MP, Forman D, Bryant H, et al. Cancer survival in Australia, Canada, Denmark, Norway, Sweden, and the UK, 1995–2007 (the International Cancer Benchmarking Partnership): an analysis of population-based cancer registry data. *The Lancet* 2011;377(9760):127-38. doi: 10.1016/s0140-6736(10)62231-3
7. Ferlay J, Parkin DM, Steliarova-Foucher E. Estimates of cancer incidence and mortality in Europe in 2008. *Eur J Cancer* 2010;46(4):765-81. doi: 10.1016/j.ejca.2009.12.014
8. Butler J, Foot C, Bomb M, et al. The International Cancer Benchmarking Partnership: an international collaboration to inform cancer policy in Australia, Canada, Denmark, Norway, Sweden and the United Kingdom. *Health policy* 2013;112(1-2):148-55. doi: 10.1016/j.healthpol.2013.03.021
9. Rubin G, Berendsen A, Crawford SM, et al. The expanding role of primary care in cancer control. *The Lancet Oncology* 2015;16(12):1231-72. doi: 10.1016/s1470-2045(15)00205-3
10. DoH. Cancer Reform Strategy. London, England: Department of Health, 2007.
11. De Angelis R, Sant M, Coleman MP, et al. Cancer survival in Europe 1999–2007 by country and age: results of EUROCARE-5—a population-based study. *The Lancet Oncology* 2014;15(1):23-34. doi: 10.1016/s1470-2045(13)70546-1
12. Richards MA. The size of the prize for earlier diagnosis of cancer in England. *British journal of cancer* 2009;101 Suppl 2:S125-9. doi: 10.1038/sj.bjc.6605402
13. Abdel-Rahman M, Stockton D, Rachet B, et al. What if cancer survival in Britain were the same as in Europe: how many deaths are avoidable? *British journal of cancer* 2009;101 Suppl 2:S115-24. doi: 10.1038/sj.bjc.6605401
14. Hiom SC. Diagnosing cancer earlier: reviewing the evidence for improving cancer survival. *British journal of cancer* 2015;112 Suppl 1:S1-5. doi: 10.1038/bjc.2015.23
15. Neal RD, Tharmanathan P, France B, et al. Is increased time to diagnosis and treatment in symptomatic cancer associated with poorer outcomes? Systematic review. *British journal of cancer* 2015;112 Suppl 1:S92-107. doi: 10.1038/bjc.2015.48
16. Chapple A, Ziebland S. Methodological and practical issues in cross-national qualitative research: Lessons from the literature and a comparative study of the experiences of people receiving a diagnosis of cancer. *Qualitative health research* 2017;[Online preview]

For peer review only

17. Simon AE, Waller J, Robb K, et al. Patient delay in presentation of possible cancer symptoms: the contribution of knowledge and attitudes in a population sample from the United kingdom. *Cancer Epidemiol Biomarkers Prev* 2010;19(9):2272-7. doi: 10.1158/1055-9965.EPI-10-0219
18. Smith LK, Pope C, Botha JL. Patients' help-seeking experiences and delay in cancer presentation: a qualitative synthesis. *The Lancet* 2005;366(9488):825-31. doi: 10.1016/s0140-6736(05)67030-4
19. Whitaker KL, Scott SE, Winstanley K, et al. Attributions of cancer 'alarm' symptoms in a community sample. *PLoS One* 2014;9(12):e114028. doi: 10.1371/journal.pone.0114028
20. Levealahti H, Tishelman C, Ohlen J. Framing the onset of lung cancer biographically: narratives of continuity and disruption. *Psycho-oncology* 2007;16(5):466-73. doi: 10.1002/pon.1080
21. Andersen B, Cacioppo JT, Roberts DC. Delay in seeking a cancer diagnosis: delay stages and psychophysiological comparison processes. *Br J Soc Psychol* 1995;34:33-52.
22. Zola I. Pathways to the doctor: from person to patient. *Social science & medicine* 1973;7:677–89.
23. Andersen RS, Paarup B, Vedsted P, et al. 'Containment' as an analytical framework for understanding patient delay: a qualitative study of cancer patients' symptom interpretation processes. *Social science & medicine* 2010;71(2):378-85. doi: 10.1016/j.socscimed.2010.03.044
24. Evans J, Chapple A, Salisbury H, et al. It can't be very important because it comes and goes": patients' accounts of intermittent symptoms preceding a pancreatic cancer diagnosis: a qualitative study. *BMJ Open* 2014;4(e004215) doi: 10.1136/bmjopen-2013004215
25. Andersen RS, Nichter M, Risør MB. Introduction: Sensations, Symptoms and Healthcare Seeking. *Anthropology in Action* 2017;24(1):1-5. doi: 10.3167/aia.2017.240101
26. Power E, Wardle J. Change in public awareness of symptoms and perceived barriers to seeing a doctor following Be Clear on Cancer campaigns in England. *British journal of cancer* 2015;112 Suppl 1:S22-6. doi: 10.1038/bjc.2015.32
27. Wyke S, Adamson J, Dixon D, et al. Consultation and illness behaviour in response to symptoms: a comparison of models from different disciplinary frameworks and suggestions for future research directions. *Social science & medicine* 2013;86:79-87. doi: 10.1016/j.socscimed.2013.03.007
28. Llanwarne N, Newbould J, Burt J, et al. Wasting the doctor's time? A video-elicitation interview study with patients in primary care. *Social science & medicine* 2017;176:113-22. doi: 10.1016/j.socscimed.2017.01.025
29. Ziebland S, Chapple A, Evans J. Barriers to shared decisions in the most serious of cancers: a qualitative study of patients with pancreatic cancer treated in the UK. *Health expectations : an international journal of public participation in health care and health policy* 2015;18(6):3302-12. doi: 10.1111/hex.12319
30. Vedsted P, Olesen F. Are the serious problems in cancer survival partly rooted in gatekeeper principles? An ecologic study. *The British journal of general practice : the journal of the Royal College of General Practitioners* 2011;61(589):e508-12. doi: 10.3399/bjgp11X588484
31. Almond SC, Summerton N. Diagnosis in General Practice. Test of time. *Bmj* 2009;338:b1878. doi: 10.1136/bmj.b1878
32. Greenhalgh T, Annandale E, Ashcroft R, et al. An open letter to The BMJ editors on qualitative research. *Bmj* 2016;352:i563. doi: 10.1136/bmj.i563

For peer review only - http://bmjopen.bmj.com/site/about/guidelines.xhtml

33. Pope C, Mays N. Qualitative Research: Reaching the parts other methods cannot reach: an introduction to qualitative methods in health and health services research. *Bmj* 1995;311(6996):42-45. doi: 10.1136/bmj.311.6996.42
34. Brown S, Castelli M, Hunter DJ, et al. How might healthcare systems influence speed of cancer diagnosis: a narrative review. *Social science & medicine* 2014;116:56-63. doi: 10.1016/j.socscimed.2014.06.030
35. OECD/EU. Health at a Glance: Europe 2016: State of Health in the EU Cycle. Paris: OECD Publishing 2016.
36. OECD. Consultations with doctors. Health at a Glance 2015: OECD Indicators. Paris: OECD Publishing 2015.
37. Wilkens J, Thulesius H, Schmidt I, et al. The 2015 National Cancer Program in Sweden: Introducing standardized care pathways in a decentralized system. *Health policy* 2016 doi: 10.1016/j.healthpol.2016.09.008
38. Vedsted P, Olesen F. A differentiated approach to referrals from general practice to support early cancer diagnosis - the Danish three-legged strategy. *British journal of cancer* 2015;112 Suppl 1:S65-9. doi: 10.1038/bjc.2015.44
39. Coyne IT. Sampling in qualitative research. Purposeful and theoretical sampling; merging or clear boundaries? *Journal of advanced nursing* 1997;26:623–30.
40. Guest G, Bunce A, Johnson L. How Many Interviews Are Enough?: An Experiment with Data Saturation and Variability. *Field Methods* 2006;18(1):59-82. doi: 10.1177/1525822x05279903
41. Chapple A, Ziebland S. Methodological and practical issues in cross-national qualitative research: Lessons from the literature and a comparative study of the experiences of people receiving a diagnosis of cancer *Qualitative health research* 2017;[In Press]
42. Pope C, Ziebland S, Mays N. Qualitative research in health care: Analysing qualitative data. *Bmj* 2000;320(7227):114-16. doi: 10.1136/bmj.320.7227.114
43. Tong A, Sainsbury P, Craig J. Consolidated criteria for reporting qualitative research (COREQ): a 32-item checklist for interviews and focus groups. *International Journal for Quality in Health Care* 2007;19(6):349–57. doi: 10.1093/intqhc/mzm042
44. Ziebland S, Coulter A, Calabrese J, et al. Understanding and Using Health Experiences: Improving patient care. Oxford: Oxford University Press 2013.
45. Renzi C, Whitaker KL, Winstanley K, et al. Unintended consequences of an 'all-clear' diagnosis for potential cancer symptoms: a nested qualitative interview study with primary care patients. *The British journal of general practice : the journal of the Royal College of General Practitioners* 2016;66(644):e158-70. doi: 10.3399/bjgp16X683845
46. Nicholson BD, Mant D, Bankhead C. Can safety-netting improve cancer detection in patients with vague symptoms? *Bmj* 2016:i5515. doi: 10.1136/bmj.i5515
47. Bankhead C, Heneghan C, Hewitson P, et al. Safety netting to improve early cancer diagnosis in primary care: development of consensus guidelines. Oxford: University of Oxford, 2011.
48. McLachlan S, Mansell G, Sanders T, et al. Symptom perceptions and help-seeking behaviour prior to lung and colorectal cancer diagnoses: a qualitative study. *Family practice* 2015;32(5):568-77. doi: 10.1093/fampra/cmv048
49. Nicholson BD, Mant D, Neal RD, et al. International variation in adherence to referral guidelines for suspected cancer: a secondary analysis of survey data. *The British journal of general practice : the journal of the Royal College of General Practitioners* 2016 doi: 10.3399/bjgp16X683449
50. NICE. Suspected cancer: recognition and referral (NG12). NICE guideline: NICE, 2015.

For peer review only

COREQ Criteria Checklist

**Domain 1: Research team and reflexivity**<br><br>*Personal Characteristics*	Covered in article?	Page
1. Interviewer/facilitator Which author/s conducted the interview or focus group?	✓	8
2. Credentials What were the researcher's credentials? E.g. PhD, MD	✓	8, 1
3. Occupation What was their occupation at the time of the study?	✓	8
4. Gender Was the researcher male or female?	✓	8, 1
5. Experience and training: What experience or training did the researcher have?	✓	8, 1
Relationship with participants		
6. Relationship established: Was a relationship established prior to study commencement?	✓	9
7. Participant knowledge of the interviewer? What did the participants know about the researcher? e.g. personal goals, reasons for doing the research	✓	9
8. Interviewer characteristics What characteristics were reported about the interviewer/facilitator? e.g. Bias, assumptions, reasons and interests in the research topic.	✓	9
**Domain 2: study design**<br><br>*Theoretical framework*		
9. Methodological orientation and Theory What methodological orientation was stated to underpin the study? e.g. grounded theory, discourse analysis, ethnography, phenomenology, content analysis.	✓	9-10
Participant selection		
10. Sampling How were participants selected? e.g. purposive, convenience, consecutive, snowball	✓	8
11. Method of approach How were participants approached? e.g. face-to-face, telephone, mail, email	✓	8-9
12. Sample size How many participants were in the study?	✓	8
13. Non-participation How many people refused to participate or dropped out? Reasons?	No participants dropped out - our distributed recruitment methods means that we do not	

For peer review only

	know how many people saw information about the study	
Setting		
14. Setting of data collection Where was the data collected? e.g. home, clinic, workplace	✓	9
15. Presence of non-participants Was anyone else present besides the participants and researchers?	✓	9
16. Description of sample What are the important characteristics of the sample? e.g. demographic data, date	✓	8, 9
Data collection		
17. Interview guide Were questions, prompts, guides provided by the authors? Was it pilot tested?	✓	9
18. Repeat interviews Were repeat interviews carried out? If yes, how many?	NA	
19. Audio/visual recording Did the research use audio or visual recording to collect the data?	✓	9
20. Field notes Were field notes made during and/or after the interview or focus group?	Researchers kept their own field diaries with notes about the interviews – these were not intended for analysis	
21. Duration What was the duration of the interviews or focus group?	✓	9
22. Data saturation Was data saturation discussed?	✓	9
23. Transcripts returned Were transcripts returned to participants for comment and/or correction?	x	
**Domain 3: analysis and findings**<br><br>*Data analysis*		
24. Number of data coders How many data coders coded the data?	✓	9-10
25. Description of the coding tree Did authors provide a description of the coding tree?	n/a for this article	
26. Derivation of themes Were themes identified in advance or derived from the data?	✓	9-10
27. Software What software, if applicable, was used to manage the data?	✓	9
28. Participant checking Did participants provide feedback on the findings?	x (But PPI did)	10
Reporting		

For peer review only

29. Quotations presented Were participant quotations presented to illustrate the themes / findings? Was each quotation identified? e.g. participant number	✓	11-17
30. Data and findings consistent Was there consistency between the data presented and the findings?	✓	11-17
31. Clarity of major themes Were major themes clearly presented in the findings?	✓	11-17
32. Clarity of minor themes Is there a description of diverse cases or discussion of minor themes?	✓	11-17

For peer review only

BMJ Open

Patients' initial steps to cancer diagnosis in Denmark, England, and Sweden: what can a qualitative, cross country comparison of narrative interviews tell us about potentially modifiable factors?

Journal:	*BMJ Open*
Manuscript ID	bmjopen-2017-018210.R2
Article Type:	Research
Date Submitted by the Author:	08-Sep-2017
Complete List of Authors:	MacArtney, John; University of Oxford, Health Experiences Research Group, Nuffield Department of Primary Care Health Sciences<br>Malmström, Marlene; Lund University and Region Skåne, The Institute for Palliative Care; Lund University, Skane University Hospital, Department of Clinical Sciences Lund, Surgery<br>Overgaard Nielsen, Trine; Aarhus Universitet, Research Centre for Cancer Diagnosis in Primary Care, Research Unit for General Practice, Department of Public Health<br>Evans, Julie; University of Oxford, Health Experiences Research Group, Nuffield Department of Primary Care Health Sciences<br>Bernhardson, Britt-Marie; Karolinska Institutet, Department of Learning, Informatics, Management and Ethics<br>Hajdarevic, Senada; Umeå University, Department of Nursing<br>Chapple, Alison; University of Oxford, Health Experiences Research Group, Nuffield Department of Primary Care Health Sciences<br>Eriksson, Lars; Karolinska Institutet, Department of Learning, Informatics, Management and Ethics; City, University of London, School of Health Sciences<br>Locock, Louise; University of Oxford, Health Experiences Research Group, Nuffield Department of Primary Care Health Sciences<br>Rasmussen, Birgit; Lund University and Region Skåne, The Institute for Palliative Care; Lunds Universitet, Department of Health Sciences<br>Vedsted, Peter; Aarhus Universitet, Research Centre for Cancer Diagnosis in Primary Care, Research Unit for General Practice, Department of Public Health; Aarhus Universitet, Department of Clinical Medicine<br>Tishelman, Carol; Karolinska Institutet, Medical Management Centre, LIME; Karolinska Universitetssjukhuset, Center for Innovation<br>Andersen, Rikke; University of Aarhus, Research Centre for Cancer Diagnosis in Primary Care, Research Unit for General Practice, Department of Public Health<br>Ziebland, Sue; University of Oxford, Health Experiences Research Group, Nuffield Department of Primary Care Health Sciences
**Primary Subject Heading**:	Health services research
Secondary Subject Heading:	Qualitative research, General practice / Family practice
Keywords:	PRIMARY CARE, QUALITATIVE RESEARCH, International health services <

	HEALTH SERVICES ADMINISTRATION & MANAGEMENT

For peer review only

Title: Patients' initial steps to cancer diagnosis in Denmark, England, and Sweden: what can a qualitative, cross country comparison of narrative interviews tell us about potentially modifiable factors?

List of authors

John I MacArtney[a], Senior Researcher.

Marlene Malmström[b, c], Senior Researcher.

Trine L Overgaard Nielsen[d], Research Assistant.

Julie Evans[a], Senior Researcher.

Britt-Marie Bernhardson[e], Senior Researcher.

Senada Hajdarevic[f], Senior Lecturer.

Alison Chapple [a], University Research Lecturer.

Lars E. Eriksson[e, g, h], Associate Professor.

Louise Locock[a], Associate Professor.

Birgit H Rasmussen[b, i], Professor.

Peter Vedsted[d, j], Professor.

Carol Tishelman[e, k], Professor.

Rikke Sand Andersen[d], Associate Professor.

Sue Ziebland[a], Professor.

[a] Health Experiences Research Group, Nuffield Department of Primary Care Health Sciences, University of Oxford, UK

[b]The Institute for Palliative Care, Lund University and Region Skåne, Sweden.

[c]Lund University, Skane University Hospital, Department of Clinical Sciences Lund, Surgery, Lund, Sweden

For peer review only

[d]Research Centre for Cancer Diagnosis in Primary Care, Research Unit of General Practice, Aarhus University, Denmark

[e]Department of Learning, Informatics, Management and Ethics, Karolinska Institutet, Sweden

[f]Department of Nursing, Umeå University, Sweden

[g]Department of Infectious Diseases, Karolinska University Hospital, Stockholm, Sweden

[h]School of Health Sciences, City, University of London, United Kingdom

[i]Lund University, Department of Health Sciences, Lund, Sweden

[j]University Clinic for Innovative Patient Pathways, Silkeborg Hospital, Department of Clinical Medicine, Aarhus University, Denmark.

[k]Center for Innovation, Karolinska University Hospital, Stockholm Sweden

Corresponding author:

John I MacArtney

Nuffield Department of Primary Care Health Sciences

University of Oxford

Radcliffe Observatory Quarter

Woodstock Road

Oxford

OX2 6GG

john.macartney@phc.ox.ac.uk

For peer review only

Abstract

Objectives

To illuminate patterns observed in International Cancer Benchmarking Programme studies by extending understanding of the various influences on presentation and referral with cancer symptoms.

Design

Cross-country comparison of Denmark, England, and Sweden with qualitative analysis of in-depth interview accounts of the pre-diagnostic process in lung or bowel cancer.

Participants

155 women and men, aged between 35 and 86 years old, diagnosed with lung or bowel cancer in the six months before interview.

Setting

Participants recruited through primary and secondary care, social media, and word-of-mouth. Interviews collected by social scientists or nurse researchers during 2015, mainly in participants' homes.

Results

Participants reported difficulties in interpreting diffuse bodily sensations and symptoms and deciding when to consult. There were examples of swift referrals by primary care professionals in all three countries. In all countries participants described difficulty deciding if and when to consult, highlighting concerns about access to GP appointments and overstretched primary care services, although this appears less prominent in the Swedish data.

For peer review only

It was not unusual for there to be more than one consultation before referral and we noted two distinct patterns of repeated consultation: (1) Situations where the participant left the primary care consultation with a plan of action about what should happen next. (2) Participants were unclear about under which conditions to return to the doctors. This second pattern sometimes extended over many weeks during which patients described uncertainty, and sometimes frustration, about if and when they should return and whether there were any other feasible investigations. The latter pattern appeared more evident in the interviews in England and Denmark than Sweden.

Conclusion

We suggest that if clear action plans, as part of safety netting, were routinely used in primary care consultations then uncertainty, false reassurance, and the inefficiency and distress of multiple consultations could be reduced.

For peer review only

Strengths and limitations of this study:

- This study provides a social science informed, qualitative cross-country comparison of 155 in-depth interviews with bowel and lung cancer patients recruited within six months of a diagnosis in Sweden, Denmark and England.
- The methods we use provide insight into why and how potentially modifiable factors identified by the International Cancer Benchmarking Project - including response to symptom experiences, differences in willingness to consult, how people negotiate access to healthcare, and what happens during consultations - affect the time to diagnosis in a primary care setting.
- The study was limited to patient interviews and no ‘first hand’ observational data were available to compare accounts.

For peer review only

Introduction

Background

No single factor is likely to explain why differences in cancer outcomes persist between high-income countries with universal health coverage. The International Cancer Benchmarking Partnership (ICBP), which compares cancer registry data and cross country surveys, has explored several hypotheses about why variations occur. These studies have shown a number of potentially modifiable factors, for example, that patterns in public knowledge about cancer awareness and beliefs were not clearly associated with variations in survival across countries.[1] The ICBP studies have also drawn attention to different patterns in public willingness to consult a Primary Care professional (PCP), with patients in the UK reporting particular concern 'not to waste the doctor's time'.[1] There are also cross country differences in general practitioners (GPs) expressed willingness to refer a patient with suspicious symptoms at their first presentation and PCPs have reported that investigations such as computerised tomography (CT) and Magnetic Resonance Imaging (MRI) were harder to access and results took longer in England and Denmark, than in Sweden.[2] There seems to be a relationship between smoking behaviour and willingness to consult: in separate, recent, studies both English [3] and Danish smokers [4] have been shown to be less likely than non-smokers to consult with red flag lung cancer symptoms. It has been suggested that this is related to the shame, blame and stigma associated with lung cancer as a smoking related disease.[5] Socio-economic deprivation, inequalities in service provision and regional differences in expectations about the likely consequences of seeking care further contribute to a complicated picture.

Denmark, England, and Sweden (all ICBP participants) were selected for this comparison because survival for lung and bowel cancer between 1995 and 2007 were persistently higher

For peer review only - http://bmjopen.bmj.com/site/about/guidelines.xhtml

in Sweden than in Denmark or England, particularly in the first year after diagnosis.[6] We chose lung and bowel cancer because they affect both genders, are the two most common causes of death from cancer across Europe,[7] their symptomatology is often diffuse or vague, and these cancers are often prone to late stage diagnosis. Earlier diagnosis has become a key healthcare research and policy target in all three countries.[8 9 10] Analyses of potentially modifiable factors have drawn attention to between country differences in stage at diagnosis and access to good care, diagnostics and screening.[11] Late presentation and long diagnostic intervals are also potentially modifiable contributory factors to poor survival.[6 11-15] Cross country comparative research can suggest routes for service redesign as well as hypotheses for further research.[16] Social and health sciences have suggested potentially modifiable factors affecting the time to diagnosis include public response to symptom experiences,[1 3-5 17-26] differences in willingness to consult, [1 3-5 17-24] how people negotiate access to healthcare,[1 18 22 27 28] what happens during consultations,[2 27 29-31] and access to diagnostic tests.[2] While surveys and cancer registry data can provide excellent high level comparative data showing patterns of association, in-depth qualitative research is needed to help illuminate why and how observed variations may occur.[32 33] We therefore designed a study to contribute a qualitative cross-country comparative analysis of narrative interviews with patients recently diagnosed with lung or bowel cancer in Denmark, England, and Sweden. In this paper the research question we ask is, what might explain some of the variations identified in the ICBP?

The healthcare systems in Denmark, England and Sweden.

All three countries have primarily tax-based health systems. However, in Sweden patient co-payments vary from no payment to 300SEK per primary care visit, depending on county council, up to a total of ~1,100SEK (£100) per year.[34] No co-payments are made in England

For peer review only - http://bmjopen.bmj.com/site/about/guidelines.xhtml

and Denmark, although there are prescription charges in all three countries. In Sweden two-thirds of GPs are publically employed, whereas in Denmark and England most are self-employed.[34] Sweden spends over 20% more on healthcare per head of population than England.[35] Contact with primary care varies between the three countries: Swedish patients consult PCPs least frequently (2.9 per annum) followed by Denmark (4.7 per annum) and UK (5.0 per annum, which includes telephone consultations).[36] In 2015 England withdrew guidance stipulating access for urgent appointments within 48 hours.[34] In some settings in Sweden, a nurse is the first person a patient speaks to when seeking an appointment.[34] Most first contact in Denmark and England is with a receptionist, who may pass the patient to a nurse to be triaged. In Denmark some receptionists have been trained to recognise potentially serious symptoms.[34] GPs have a central role in making referrals to lung and colorectal specialists in all three countries [34] The national cancer plan in Sweden is regionally administered and focused on secondary care[37], whereas in England and Denmark specific clinical guidance and targets for PCPs are also included.[34] England (implemented 2006) and Denmark (implemented 2014) have national cancer screening programmes for bowel cancer. In Sweden bowel screening was implemented regionally, with some regions having some form of programme from 2008. Currently it is recommended for 60-74 year olds, although this again varies by region. Denmark has increased rapid access clinics and access to investigations, while in England referral management systems have been interspersed between the GP and secondary care.[34 38]

Methods

Sampling and recruitment

We interviewed 155 people during 2015 who were within six months of a diagnosis of lung or bowel cancer in the three countries. Purposive sampling[39] within each country was used to

For peer review only

achieve gender balance and variation across age, urban and rural locations, and pathway to diagnosis (see Table 1). An experienced sociologist in England (JM) and an anthropologist in Denmark (TON) recruited and conducted all the interviews in their countries, while three experienced nurse researchers collaborated in different regions of Sweden (BMB, SH, and MM). Participants were initially approached by treating clinicians in hospital clinics in all three countries. To reach data saturation, in England and Denmark this approach was supplemented with some additional recruitment from support groups, social media, and word-of-mouth. All potential participants were provided with an information sheet that included the rationale for the study and an explanation of what would be involved if they consented to take part in the study. Data saturation[40] was judged to have been reached in the analytic categories for all three countries before recruitment closed.

Data collection

Interviews took place in participants' homes, unless they preferred another location. One-to-one interviews were preferred, but a small number of participants requested a non-participating family member to be present. Interviews started with an open ended question: "Could you start by telling me, in your own words and in as much detail as you want, about everything that has happened since you first started to suspect there might be a problem with your health?" Interviews lasted between 45 minutes to 1.5 hours. During the interview the researchers used a semi-structured topic guide based on social science theories, and the cancer research literature (highlighted in the Introduction), including factors related to the diagnostic interval. [1-5 17-31] The research team had extensive discussions about the topics to ensure comparable data were collected.[41] Interviews were audio recorded and transcribed verbatim.

For peer review only - http://bmjopen.bmj.com/site/about/guidelines.xhtml

Analysis

Monthly teleconferences with the field research team (all of whom had a high level of spoken and written English) were held throughout the development of the topic guide, recruitment, data collection and analysis phases. Interview accounts were analysed for narrative themes that structured participant experiences. To do this, in each country interview transcripts were imported into specialist computer software (NVivo 10) for organising textual data and were coded by the interviewing researchers. The three research teams conducted separate thematic analysis with their own data using a coding frame developed through discussion in the teleconferences and based on the (anticipated) themes from the topic guide and on emergent themes.[42] One two-day and one four-day analysis and writing workshops were held with the research teams from all three countries. Anticipated issues drew on existing knowledge and theoretical insights.[1-5 17-31] Emerging analyses were iteratively tested within and between datasets. Allocations between the analysis categories (including identifying participants who described leaving the PCP consultation with an understanding of what should happen next and those who reported uncertainty about what to do) was discussed among the field researchers. Direct quotes from the interviews (translated into English by the bi-lingual researchers) are used to illustrate the results. Analysis and findings are presented in accordance with relevant COREQ[43] criteria for reporting qualitative data and are consistent with interpretive approaches to analysis.[44]

Patient involvement

Public and Patient Involvement (PPI) was conducted in accordance with good practice in each country. Representatives in England with experience of bowel or lung cancer were involved in preparing the funding application, the study design and the multidisciplinary advisory group, as well as advising on recruitment strategies, including the patient

For peer review only - http://bmjopen.bmj.com/site/about/guidelines.xhtml

information sheets. In all three countries PPI members were invited to comment on the draft interview topic guide. A group of three English PPI members met with the research team to look at a subset of the English data. Drafts of study papers have been circulated to the advisory panel. Summaries of the main study findings, prepared for a stakeholder final event in 2017, will be circulated to all study participants who expressed interest in seeing the results.

Results

We compared the three sets of interviews to consider the following potentially modifiable steps, drawn from the literature, on the pre-diagnosis pathway: people's initial responses to bodily sensations and the transformation of these into symptoms justifying medical advice; accounts of accessing medical help; what happened in the consultation; and, planning for follow-up.

		Denmark		England		Sweden		Percentages	
		BC	LC	BC	LC	BC	LC	BC	LC
**Number of participants**		28	22	25	20	30	30	54%	46%
**Female**		13	8	12	10	14	15	47%	46%
**Age range**	**31-50**	2	0	4	*2*	2	2	10%	5%
	**51-70**	19	15	13	12	14	21	55%	67%
	**71-90**	7	7	8	6	14	7	35%	28%

Table 1: Participant demographic characteristics across the three countries

For peer review only - http://bmjopen.bmj.com/site/about/guidelines.xhtml

Awareness and responses to signs and symptoms

The first potentially modifiable factors are the type of sensations and bodily changes people considered to justify seeking help. Participants in all three countries described considerable uncertainty about the possible implications of bodily sensations and when or if they should be considered symptoms warranting medical advice, especially if they felt well. For example, apart from some blood in his stools a participant in England said he had been "fit as anything and not feeling ill at all" (England-BC18). Even those who looked up their symptoms and risk factors online were reassured if they found that relatively few items on the checklists applied to them. People reasoned that they must be low risk if they felt they had a good diet, were physically active, non-smokers (or long term ex-smokers), generally fit, relatively young, or without a family history of cancer. This reasoning suggested that some patients expected there to be a linear relationship between healthy input and outcomes. For example, a participant in Denmark who acknowledged "[I] smoke and [drink] alcohol and [eat] too much meat…" went on to suggest that other behaviours offered some protection from the profile of a typical cancer patient:

> Denmark-BC01: I have read about this, I'm very much into organic food. All the things you say can cause cancer… you don't know […]. I have at least lived a healthy life with vegetables everyday…and fruit, so maybe I'm not the typical cancer patient.

Persistent coughs, pains, stomach aches, changes in bowel habits, bleeding or constipation were attributed to everyday causes or to more familiar diseases such as influenza, pneumonia, Irritable Bowel Syndrome (IBS) or hemorrhoids.[21] People with lung cancer, who smoked cigarettes, reasoned that a persistent cough was a 'normal' bodily response to smoking. Thus, even those who are aware that they have risk factors, or suspicious symptoms, may conclude that overall their risk is low and not seek medical help. Consistent with Zola's classic work

on triggers to the consultation, when people *did* consult this was usually because the symptoms: interfered with daily life; became evident to others who encouraged them to consult; or, exceeded a self-imposed time limit[22], rather than because they suspected that they might have cancer.

Access to primary care

The second potentially modifiable factor is whether the participant felt able to access primary care. There were some differences apparent in accounts from the three countries. In England, people talked about long waiting times for 'non-urgent' appointments or (echoing ICBP findings) said they were reluctant to trouble busy GPs with potentially minor illnesses that might be treatable with over-the counter remedies.[1] A participant in England, later diagnosed with bowel cancer, had concluded, "It's haemorrhoids, leave it. Doctors are busy with other things" (England-BC20). Another participant in England with lung cancer explained why she hadn't consulted with a cough:

> England-LC12: When you hear that the GPs are so busy and being bothered by, you know, minor illnesses it makes you reluctant to go with something that you think [is minor] . . . I was, well, "What can the doctor do? They won't want to give you antibiotics for a virus." That was what I, I thought. And I just didn't want to really bother the doctor.

A participant in Denmark similarly reflected upon an implication of GPs' time pressures:

> Denmark-LC15: The system is not always that easy. First you have to convince the secretary, that you need an appointment, right. That is what happens when they are too busy.

For peer review only - http://bmjopen.bmj.com/site/about/guidelines.xhtml

In contrast, we were struck that accounts in Sweden rarely included detailed justifications for using the doctor's time, nor did they suggest that the onus was on the participant to determine whether the matter was serious, or urgent. For example, a participant in Sweden diagnosed with bowel cancer said:

> Sweden-BC051: It began like this, that I found a little blood ... I can say in the stool, very little . . . And I tend to ignore such things ... but this was just strange enough, so I thought, "no, I've got to go to the health centre and hear what it is". And so I did.

Another participant in Sweden with lung cancer said, "It's better to go one time too many than one too few" (Sweden-LC102). This sentiment was not expressed in any of our interviews in England.

During the consultation

The third potentially modifiable factor is what happens when the patient presents with symptoms. Seeking healthcare is not just a matter of making an appointment, but also of being able to present one's problem and be heard.[9] In all three countries there were examples of a single consultation and a prompt referral. A participant in England said,

> England-BC18: Our local GP was brilliant, she said, "I'm not gonna do the kind of normal give you this stool sample packet, all that kinda stuff. I'm gonna hotline you straight to [hospital] down the road there for an endoscopy. Skip all the intermediate stuff and go straight into endoscopy", which was a fabulous decision, if you think of it.

Similarly a participant in Denmark explained,

For peer review only - http://bmjopen.bmj.com/site/about/guidelines.xhtml

> Denmark-BC13: The stomach pains had been going on for a while, but one day I just could not stand it anymore and drove straight down to my doctor's office. My doctor examined it and immediately said: "I will send you off to the hospital". And I was [sent off] in the afternoon. That was quick.

A participant in Sweden with bowel cancer recalled the GP saying, "No, I cannot see anything, but for safety's sake I will send you to a specialist […] so they get a closer look at it" (Sweden-BC051).

There were also examples of participants in all three countries who told us that they had returned to the doctors over periods of weeks or even months, before they were referred for further investigations. Some participants in England and Denmark diagnosed with lung cancer said they had been repeatedly treated with antibiotics for chest infections. Participants diagnosed with bowel cancer from all three countries described inconclusive consultations for presumed constipation, haemorrhoids, IBS, Crohn's, or psychological causes; however in our data this appeared more often in interviews from Denmark and England. This example from England typifies this:

> England-BC09: . . . each time it was treated obviously as constipation. There was never even any mention of anything else. . . He's (the GP) a very, "I can do it. I can fix it. I can do it". But each time I come away, it was so sort of disheartening, because it seemed to be about what he could do and not actually helping the situation. But I did have, I had all sorts of laxatives from him. It's as if like we just keep trying to do this, but I was in agony each time I took it. I did explain that each time. . . . But, yeah, nothing really. It was more like the repetitive sort of appointment.

For peer review only - http://bmjopen.bmj.com/site/about/guidelines.xhtml

Making plans for follow-up

The fourth potentially modifiable factor is what happens after those consultations which do not include a referral. We looked in detail at the accounts of those participants who reported two or more presentations before referral and noted two different types of experience, depending on whether or not the patient reported leaving the consultation knowing what to do next.

<u>Repeated consultation(s) with patient awareness of next steps</u>

First, there were participants in all three countries who described leaving the consultation with an understanding about what to expect, including how long to wait for symptoms to abate, what should prompt them to return, and what would happen after that. For example this participant in Sweden said,

> Sweden-LC129: And then the [GP] said, "No it's nothing. Your lungs sound very healthy, but you've had this cough for the last eight weeks and if it does not disappear in another week, then please call us. Then we'll see."

The participants in this 'planned' group had also known what would be likely to happen if and when they did return; all three countries included examples of people who knew that further treatments or investigations would be needed if the symptoms did not resolve:

> England-LC17: And he [GP] prescribed two weeks antibiotics. And then he said that after that, if it didn't make any difference then he might prescribe omeprazole and maybe then a chest x-ray.

Follow-up advice can be reinforced by practice staff when delivering a normal test result in the course of their diagnostic investigations.[45] For example a participant in Sweden reported

that the practice nurse said, "if [it] gets worse – for example if you lose weight – contact us directly" (Sweden-BC055).

Repeat consultation(s) with patient uncertainty about next steps

Second, there were participants who were unsure what would or *should* happen next, such as England-BC09 quoted above who described a "disheartening" and "repetitive sort of appointment". These participants included those who worried that it was not appropriate to return to the doctor with the same symptoms and those who had been reassured at the initial consultation. The following participant in Denmark had requested a colonoscopy after many months of unusual symptoms and several investigations for other possible causes.

> Denmark-BC05: I can see now that I had these symptoms for three years. They [the doctors] thought that it was only the myalgia. But it probably was the colon cancer, even though I did not have the usual symptoms, you know. No bleeding, or diarrhoea or constipation. Just this strange heaviness in my lower parts. So I had an ultrasound of my bladder and kidneys, an x-ray of my lumbar area… And then I finally asked for a colonscopy. My doctor asked me if I was sure… of course I was… but I had not thought of cancer.

In these accounts people had been unsure what to expect and left without a plan about what to do next. Unsurprisingly, people who had presented on two, three or more occasions described some frustration. In retrospect, some wondered if the PCP had decided that a "test of time" or "wait-and-see" was the best course of action, without sharing their reasoning.[30 31]

> England-BC03: And the simple thing and I agree the most obvious thing was, you know, piles and irritable bowel syndrome . . . I guess they, the thought was, "Well

For peer review only - http://bmjopen.bmj.com/site/about/guidelines.xhtml

> we'll send her away with that for now and if doesn't work, she's bound to come back."

Accounts of repeated consultations, without an action plan communicated to the patient, featured more in the interviews with patients in England and Denmark than in Sweden. Patients may welcome reassurance from their PCP about their suspicious symptoms, and this may not be a problem if the practitioner takes responsibility for following up the patient as part of safety netting – the process of communicating with patient what to expect, documenting any action plans, and following the patient up as agreed.[46] A Swedish lung cancer patient explained that the GP had telephoned to see 'how she was' after a gastroscopy and made an urgent referral:

> Sweden-LC123 I got a referral to ultrasound of my thyroid and upper gastro. And then they discovered in a gastroscopy that I had oesophagitis . . . [a week later the GP] called to ask how I was. I said, "now it is bad", I said. "I think I have to go to the hospital urgently as it is now, it's ... I cannot breathe". "Come here", she said, "and we'll make an urgent referral".

Discussion

Principal findings

Our international qualitative comparison has considered four potentially modifiable features of the pre-diagnostic experience of lung and bowel cancer patients. Across the countries there are many similarities in how symptoms were experienced and in awareness of what the symptoms might mean. Our analysis suggests differences between countries in people's accounts of their willingness to consult and re-consult, their descriptions of what happened during consultations and, for those who did not receive an immediate referral, their confusion or clarity about what they should look for, when to return and what to expect. Reluctance and

For peer review only - http://bmjopen.bmj.com/site/about/guidelines.xhtml

practical barriers to consulting the GP, and uncertainty about whether and when to re-attend after an initial consultation, appeared more evident in interviews in Denmark and England. This indicates the benefit of communication of clear action plans to patients, including information on symptom development, and other safety netting approaches,[47] which could well be developed more. PCPs action plans might include a 'test of time', yet this reasoning should be clearly communicated to the patient. This could also mean that patients would receive more timely referrals, and avoid the repeated visits to primary care which featured particularly in English and Danish interviews.[31]

Strengths and weaknesses of the study

The study design – a cross-cultural comparison using in-depth qualitative interview – is the first of its kind to study the route to cancer diagnosis. Our findings suggest potentially modifiable factors that could improve timely diagnosis of cancer in all three countries, although the scope for improvement may be greater in England and Denmark.

This qualitative study was designed to illuminate findings from the ICBP studies and is intended to be indicative rather than conclusive. Findings extend observations from other research that highlights the centrality of the primary care encounter in pre-diagnostic experiences. Participants' cancer disease stage is likely to have affected their experiences. To achieve diversity we recruited people who were diagnosed with very early stage and others diagnosed with advanced disease in all three countries. Participants who were recruited via social media in Denmark and England may also be thought to have had different experiences, than those recruited via clinics. All participants were, or had been, under specialist cancer care and within six months of diagnosis. Each data set was analysed by the country specific

For peer review only

team, thus benefitting from familiarity with healthcare context, language and culture. These country specific analyses were shared as part of a careful collaborative process, involving workshops, teleconferences and several drafts and further targeted analyses. Along with the teleconferences, these meetings helped to mitigate any potential for differences in the data arising from differences in country or disciplinary approaches to interviewing or analysis.[41]

Strengths and weaknesses in relation to other studies

This study was designed to explore whether a cross-country comparison of qualitative interviews could help to illuminate findings from the ICBP survival analyses and surveys. While the high level comparisons of large quantitative data bases and surveys provide invaluable information, qualitative studies can suggest why these patterns may occur and identify avenues for service redesign, such as action plans, some of which could be implemented without further research. We did not observe the consultations described by the patients and it is possible that the PCPs involved did try to communicate an action plan, even if it did not feature in the patient's account. Again, we have no reason to suspect that variations in recall would differ between the countries. It is well known that public knowledge of cancer symptoms is insufficient to prompt people to seek help, as recently demonstrated in ICBP surveys.[1] Our work was guided by insights from the social and healthcare sciences into the complexity inherent in decisions to consult. These are influenced by people's ideas about their own candidacy, related to family history and health behaviours, as well as their access to care.[25 28 48] Evans et al have shown that patients, and their doctors, may be falsely reassured if a symptom comes and goes, reasoning that intermittent symptoms are unlikely to be serious.[24] Renzi and colleagues have drawn attention to the potential negative consequences of reassurance through an 'all-clear' result from investigations, which could be given with or without an encouragement to return.[45]

For peer review only - http://bmjopen.bmj.com/site/about/guidelines.xhtml

This study provides further insight into ICBP findings of how cancer symptoms are managed in primary care, in particular how delays may be compounded when a patient is not referred promptly and no clear plan is communicated about the circumstances in which they should return for further consultation or investigations.[2 49] Current research is exploring how and why planning, communicating action plans to patients, and following-up are operationalised as part of safety netting in English primary care and what approaches are acceptable to both GPs and patients.[46]

Implications for clinicians and policy makers

There are several important differences between these three countries, for example in access to secondary care, in spending on health services and in the numbers of licensed practitioners. The health system in England has less money, fewer doctors and nurses and fewer hospital beds then Sweden or Denmark.[34-36] These factors may yet prove critical in limiting improvements in cancer survival. Patients in Sweden consult their GP less often than patients in England or Denmark, but for longer.[36] Shorter consultations may not be efficient – patients in England consult their GPs for nearly one hour per year, yet spread over five appointments while, on average, patients in Sweden visit primary care twice a year, but for a mean of 20 minutes per appointment.

Through our international comparison we have been able to identify opportunities for pathway and service improvement that could promote earlier stage diagnosis. The perception of systemic barriers (e.g. access to PCP appointments) may prevent patients seeking timely help, even in the presence of potentially alarming symptoms (e.g. blood in stools or a cough that lasts for three weeks). While awareness of symptoms may be important, there is a limit to

For peer review only

how much effect increasing public awareness could have in settings where access is limited and people are uncertain when to consult, or re-consult.[23 26]

The importance of safety netting was emphasised in the 2015 guidance for suspected cancer from the UK National Institute for Health and Care Excellence[50] and a 2011 Delphi study of British GPs and primary care researchers led to publication of suggestions for how to make safety netting more effective.[46] In Denmark, direct access to investigation from general practice, with the aim to lower the GPs' threshold for referring and lengthy 'wait-and-see' approaches[30], is being investigated along with a focus on safety-netting. Our findings suggest that clearly communicated action plans at the end of *any* primary care consultation could be encouraged, without the need for further research. This would be likely to improve the patient's experience and may also reduce demand for repeated appointments and diagnostic delay.

Unanswered questions/need for further research

Research is needed into ways of redesigning primary care services in all three countries to reduce (perceived) barriers to consultation. NICE has recommended that British PCPs employ safety-netting to reduce delays in the diagnosis of cancer, but there has been little research to understand what constitutes best practice. Further research might address how a "test of time" or "wait-and-see" approach that includes clear planning affects the number of follow-up visits and cancer stage at diagnosis. We do not know how action planning is communicated in the consultation, nor what effect this may have on the length or frequency of consultations. This could be explored in future mixed methods research, ideally including conversation analysis of the consultation. Cross country differences in referral and follow up practices in lung cancer and bowel cancer patients could be investigated further, especially in

For peer review only

the light of recent studies suggesting that smokers (in Denmark and England) may be less likely to consult. Is the same pattern apparent in Sweden – and if not, why not?

Conclusion

If all primary care consultations concluded with a clear description of what should happen next and the circumstances in which the patient should return, this might avert some of the uncertainty and repeated consultations that were described in these interviews. We conclude that there is an opportunity for clearer communication and better follow-up strategies in primary care – neither of which will put additional pressure on specialist services, yet hold the potential to reduce diagnostic delays with few additional resources.

For peer review only

Other BMJ requirements

Acknowledgements

We wish to thank Professor Mike Richards (then National Cancer Director) and Professor Chris Ham (Chief Executive King's Fund), who had the original idea for the study as well as facilitated bringing the three research teams together. We are extremely grateful to the people who took part in this research in all three countries, and to the study advisory panel, including patient and public representatives, who helped design the study and provided comments on an earlier draft of this manuscript. We also acknowledge the support of the National Institute for Health Research, through the Clinical Research Network, who helped recruit patients into the English arm of the study. We would also like to thank all those who helped to recruit participants: In England we would like to thank the NHS Hospital Trusts that assisted with this study and Patients Active in Research: Thames Valley, along with the following charities who posted links or circulated our details on social media: Beating Bowel Cancer, Bowel Cancer UK, Roy Castle Lung Cancer Foundation, British Lung Foundation, and healthtalk.org. In Denmark we want to thank the Lung Cancer Patient organisation, as well as the Colon Cancer Patient organisation for assisting us in recruiting patients. We also wish to thank the local support groups of the national Danish Cancer Society, as well as the local oncology departments in various regions of Denmark. In Sweden we would like to thank the nurses and physicians who helped with recruitment.

For peer review only

Author Contributions

SZ developed the idea for the manuscript. JM wrote the first draft, with edits made by SZ, JE, MM, CT, RSA and PV. BMB, AC, LE, SH, LL, TLON, AC, and BR contributed to the analysis meetings and provided comments on drafts.

For peer review only - http://bmjopen.bmj.com/site/about/guidelines.xhtml

Competing Interests

All authors have completed the ICMJE uniform disclosure form at www.icmje.org/coi_disclosure.pdf and declare: no support from any organisation for the submitted work; no financial relationships with any organisations that might have an interest in the submitted work in the previous three years; no other relationships or activities that could appear to have influenced the submitted work.

Copyright Statement

The Corresponding Author has the right to grant on behalf of all authors and does grant on behalf of all authors, a worldwide licence to the Publishers and its licensees in perpetuity, in all forms, formats and media (whether known now or created in the future), to i) publish, reproduce, distribute, display and store the Contribution, ii) translate the Contribution into other languages, create adaptations, reprints, include within collections and create summaries, extracts and/or, abstracts of the Contribution, iii) create any other derivative work(s) based on the Contribution, iv) to exploit all subsidiary rights in the Contribution, v) the inclusion of electronic links from the Contribution to third party material where-ever it may be located; and, vi) licence any third party to do any or all of the above.

Ethics

Approvals for research ethics and governance were obtained separately according to the requirements in each country as follows:

England: Research Ethics Service reference 14/NS/1035

Denmark: The Biomedical Research Ethics Committee System Act does not apply to this project, as the project does not implicate the use of human biological materials. Standard ethical protocol according to the American Anthropological Association was followed.

For peer review only

Sweden: Regional Ethics Board, Lund, Sweden, reg. no 2014/819

Informed consent

All participants signed a consent form before taking part in an interview. Participants in England signed a second form after interview giving permission for their interview to be available to researchers at the University of Oxford, and other universities under licence from Oxford, for use in research, teaching, and other publications.

Funding statement and independence of the researchers

This paper presents independent research funded by organisations from 3 European countries as follows:

In the UK, the study was supported by the National Awareness and Early Diagnosis Initiative (NAEDI), http://www.naedi.org.uk. The contributing partners include: Cancer Research UK; Department of Health, England; Economic and Social Research Council; Health and Social Care Research and Development Division, Pubic Health Agency, Northern Ireland; National Institute for Social Care and Health Research, Wales; and the Scottish Government. This funding also covered the costs associated with the comparative analysis meetings in Denmark and Sweden and funded translation of the Danish and Swedish material for publications.

During the study LL was Director of Applied Research at the Health Experiences Research Group, Nuffield Department of Primary Care Health Sciences, University of Oxford, and was supported by the National Institute for Health Research (NIHR) Oxford Biomedical Research Centre and the NIHR Collaboration for Leadership in Applied Health Research and Care Oxford (CLAHRC) at Oxford Health NHS Foundation Trust.

For peer review only

In Denmark, the study was supported by the Research Centre for Cancer Diagnosis in Primary Care funded by The Danish Cancer Society and the Novo Nordic Foundation.

In Sweden, the study was supported by the Vårdal Foundation; the Strategic Research Program in Care Sciences (SFO-V), Umeå University; the Cancer Research Foundation in Northern Sweden; and from government funding of clinical research within the National Health Service, Sweden.

The views expressed in this paper are those of the authors, and not necessarily those of the NAEDI, Danish and Swedish funding partners.

Role of the sponsor

The study sponsor(s) or funder(s), had no role in the study design; the collection, analysis, and interpretation of data; writing of the report; or in the decision to submit the article for publication.

Access of researchers to all the data

All researchers in each country had access to all the data gathered in their own country. Interview transcripts were not translated and were not shared between countries, but Swedish and Danish interview extracts were translated by the bi-lingual researchers for the analytic discussions. The team lead in each country (SZ, RSA and BR) takes responsibility for the integrity of the data and the accuracy of the data analysis.

Transparency declaration

For peer review only

The lead author (the manuscript's guarantor) affirms that the manuscript is an honest, accurate, and transparent account of the study being reported; that no important aspects of the study have been omitted; and that any discrepancies from the study as planned (and, if relevant, registered) have been explained.

Data sharing statement

In Denmark the data are available for secondary analysis in conjunction with members of the research group named Comparative Cancer Experiences.

In England the participants gave informed consent for data to be copyrighted to the University of Oxford for secondary analysis, broadcasting, publication and teaching.

In Sweden the data are available for secondary analysis in conjunction with members of the research group named Comparative Cancer Experiences.

For peer review only

References

1. Forbes LJ, Simon AE, Warburton F, et al. Differences in cancer awareness and beliefs between Australia, Canada, Denmark, Norway, Sweden and the UK (the International Cancer Benchmarking Partnership): do they contribute to differences in cancer survival? *British journal of cancer* 2013;108(2):292-300. doi: 10.1038/bjc.2012.542
2. Rose PW, Rubin G, Perera-Salazar R, et al. Explaining variation in cancer survival between 11 jurisdictions in the International Cancer Benchmarking Partnership: a primary care vignette survey. *BMJ Open* 2015;5(5):e007212. doi: 10.1136/bmjopen-2014-007212
3. Friedemann Smith C, Whitaker KL, Winstanley K, et al. Smokers are less likely than non-smokers to seek help for a lung cancer 'alarm' symptom. *Thorax* 2016;71:659-61.
4. Sele LM, Elnegaard S, Balasubramaniam K, et al. Lifestyle factors and contact to general practice with respiratory alarm symptoms-a population-based study. *BMC Fam Pract* 2016;17:47. doi: 10.1186/s12875-016-0444-9
5. Chapple A, Ziebland S, McPherson A. Stigma, shame, and blame experienced by patients with lung cancer: qualitative study. *Bmj* 2004;328(7454):1470. doi: 10.1136/bmj.38111.639734.7C
6. Coleman MP, Forman D, Bryant H, et al. Cancer survival in Australia, Canada, Denmark, Norway, Sweden, and the UK, 1995–2007 (the International Cancer Benchmarking Partnership): an analysis of population-based cancer registry data. *The Lancet* 2011;377(9760):127-38. doi: 10.1016/s0140-6736(10)62231-3
7. Ferlay J, Parkin DM, Steliarova-Foucher E. Estimates of cancer incidence and mortality in Europe in 2008. *Eur J Cancer* 2010;46(4):765-81. doi: 10.1016/j.ejca.2009.12.014
8. Butler J, Foot C, Bomb M, et al. The International Cancer Benchmarking Partnership: an international collaboration to inform cancer policy in Australia, Canada, Denmark, Norway, Sweden and the United Kingdom. *Health policy* 2013;112(1-2):148-55. doi: 10.1016/j.healthpol.2013.03.021
9. Rubin G, Berendsen A, Crawford SM, et al. The expanding role of primary care in cancer control. *The Lancet Oncology* 2015;16(12):1231-72. doi: 10.1016/s1470-2045(15)00205-3
10. DoH. Cancer Reform Strategy. London, England: Department of Health, 2007.
11. De Angelis R, Sant M, Coleman MP, et al. Cancer survival in Europe 1999–2007 by country and age: results of EUROCARE-5—a population-based study. *The Lancet Oncology* 2014;15(1):23-34. doi: 10.1016/s1470-2045(13)70546-1
12. Richards MA. The size of the prize for earlier diagnosis of cancer in England. *British journal of cancer* 2009;101 Suppl 2:S125-9. doi: 10.1038/sj.bjc.6605402
13. Abdel-Rahman M, Stockton D, Rachet B, et al. What if cancer survival in Britain were the same as in Europe: how many deaths are avoidable? *British journal of cancer* 2009;101 Suppl 2:S115-24. doi: 10.1038/sj.bjc.6605401
14. Hiom SC. Diagnosing cancer earlier: reviewing the evidence for improving cancer survival. *British journal of cancer* 2015;112 Suppl 1:S1-5. doi: 10.1038/bjc.2015.23
15. Neal RD, Tharmanathan P, France B, et al. Is increased time to diagnosis and treatment in symptomatic cancer associated with poorer outcomes? Systematic review. *British journal of cancer* 2015;112 Suppl 1:S92-107. doi: 10.1038/bjc.2015.48
16. Chapple A, Ziebland S. Methodological and practical issues in cross-national qualitative research: Lessons from the literature and a comparative study of the experiences of people receiving a diagnosis of cancer. *Qualitative health research* 2017;[Online preview]

For peer review only - http://bmjopen.bmj.com/site/about/guidelines.xhtml

17. Simon AE, Waller J, Robb K, et al. Patient delay in presentation of possible cancer symptoms: the contribution of knowledge and attitudes in a population sample from the United kingdom. *Cancer Epidemiol Biomarkers Prev* 2010;19(9):2272-7. doi: 10.1158/1055-9965.EPI-10-0219
18. Smith LK, Pope C, Botha JL. Patients' help-seeking experiences and delay in cancer presentation: a qualitative synthesis. *The Lancet* 2005;366(9488):825-31. doi: 10.1016/s0140-6736(05)67030-4
19. Whitaker KL, Scott SE, Winstanley K, et al. Attributions of cancer 'alarm' symptoms in a community sample. *PLoS One* 2014;9(12):e114028. doi: 10.1371/journal.pone.0114028
20. Levealahti H, Tishelman C, Ohlen J. Framing the onset of lung cancer biographically: narratives of continuity and disruption. *Psycho-oncology* 2007;16(5):466-73. doi: 10.1002/pon.1080
21. Andersen B, Cacioppo JT, Roberts DC. Delay in seeking a cancer diagnosis: delay stages and psychophysiological comparison processes. *Br J Soc Psychol* 1995;34:33-52.
22. Zola I. Pathways to the doctor: from person to patient. *Social science & medicine* 1973;7:677–89.
23. Andersen RS, Paarup B, Vedsted P, et al. 'Containment' as an analytical framework for understanding patient delay: a qualitative study of cancer patients' symptom interpretation processes. *Social science & medicine* 2010;71(2):378-85. doi: 10.1016/j.socscimed.2010.03.044
24. Evans J, Chapple A, Salisbury H, et al. It can't be very important because it comes and goes": patients' accounts of intermittent symptoms preceding a pancreatic cancer diagnosis: a qualitative study. *BMJ Open* 2014;4(e004215) doi: 10.1136/bmjopen-2013004215
25. Andersen RS, Nichter M, Risør MB. Introduction: Sensations, Symptoms and Healthcare Seeking. *Anthropology in Action* 2017;24(1):1-5. doi: 10.3167/aia.2017.240101
26. Power E, Wardle J. Change in public awareness of symptoms and perceived barriers to seeing a doctor following Be Clear on Cancer campaigns in England. *British journal of cancer* 2015;112 Suppl 1:S22-6. doi: 10.1038/bjc.2015.32
27. Wyke S, Adamson J, Dixon D, et al. Consultation and illness behaviour in response to symptoms: a comparison of models from different disciplinary frameworks and suggestions for future research directions. *Social science & medicine* 2013;86:79-87. doi: 10.1016/j.socscimed.2013.03.007
28. Llanwarne N, Newbould J, Burt J, et al. Wasting the doctor's time? A video-elicitation interview study with patients in primary care. *Social science & medicine* 2017;176:113-22. doi: 10.1016/j.socscimed.2017.01.025
29. Ziebland S, Chapple A, Evans J. Barriers to shared decisions in the most serious of cancers: a qualitative study of patients with pancreatic cancer treated in the UK. *Health expectations : an international journal of public participation in health care and health policy* 2015;18(6):3302-12. doi: 10.1111/hex.12319
30. Vedsted P, Olesen F. Are the serious problems in cancer survival partly rooted in gatekeeper principles? An ecologic study. *The British journal of general practice : the journal of the Royal College of General Practitioners* 2011;61(589):e508-12. doi: 10.3399/bjgp11X588484
31. Almond SC, Summerton N. Diagnosis in General Practice. Test of time. *Bmj* 2009;338:b1878. doi: 10.1136/bmj.b1878
32. Greenhalgh T, Annandale E, Ashcroft R, et al. An open letter to The BMJ editors on qualitative research. *Bmj* 2016;352:i563. doi: 10.1136/bmj.i563

For peer review only

33. Pope C, Mays N. Qualitative Research: Reaching the parts other methods cannot reach: an introduction to qualitative methods in health and health services research. *Bmj* 1995;311(6996):42-45. doi: 10.1136/bmj.311.6996.42
34. Brown S, Castelli M, Hunter DJ, et al. How might healthcare systems influence speed of cancer diagnosis: a narrative review. *Social science & medicine* 2014;116:56-63. doi: 10.1016/j.socscimed.2014.06.030
35. OECD/EU. Health at a Glance: Europe 2016: State of Health in the EU Cycle. Paris: OECD Publishing 2016.
36. OECD. Consultations with doctors. Health at a Glance 2015: OECD Indicators. Paris: OECD Publishing 2015.
37. Wilkens J, Thulesius H, Schmidt I, et al. The 2015 National Cancer Program in Sweden: Introducing standardized care pathways in a decentralized system. *Health policy* 2016 doi: 10.1016/j.healthpol.2016.09.008
38. Vedsted P, Olesen F. A differentiated approach to referrals from general practice to support early cancer diagnosis - the Danish three-legged strategy. *British journal of cancer* 2015;112 Suppl 1:S65-9. doi: 10.1038/bjc.2015.44
39. Coyne IT. Sampling in qualitative research. Purposeful and theoretical sampling; merging or clear boundaries? *Journal of advanced nursing* 1997;26:623–30.
40. Guest G, Bunce A, Johnson L. How Many Interviews Are Enough?: An Experiment with Data Saturation and Variability. *Field Methods* 2006;18(1):59-82. doi: 10.1177/1525822x05279903
41. Chapple A, Ziebland S. Methodological and practical issues in cross-national qualitative research: Lessons from the literature and a comparative study of the experiences of people receiving a diagnosis of cancer *Qualitative health research* 2017;[In Press]
42. Pope C, Ziebland S, Mays N. Qualitative research in health care: Analysing qualitative data. *Bmj* 2000;320(7227):114-16. doi: 10.1136/bmj.320.7227.114
43. Tong A, Sainsbury P, Craig J. Consolidated criteria for reporting qualitative research (COREQ): a 32-item checklist for interviews and focus groups. *International Journal for Quality in Health Care* 2007;19(6):349–57. doi: 10.1093/intqhc/mzm042
44. Ziebland S, Coulter A, Calabrese J, et al. Understanding and Using Health Experiences: Improving patient care. Oxford: Oxford University Press 2013.
45. Renzi C, Whitaker KL, Winstanley K, et al. Unintended consequences of an 'all-clear' diagnosis for potential cancer symptoms: a nested qualitative interview study with primary care patients. *The British journal of general practice : the journal of the Royal College of General Practitioners* 2016;66(644):e158-70. doi: 10.3399/bjgp16X683845
46. Nicholson BD, Mant D, Bankhead C. Can safety-netting improve cancer detection in patients with vague symptoms? *Bmj* 2016:i5515. doi: 10.1136/bmj.i5515
47. Bankhead C, Heneghan C, Hewitson P, et al. Safety netting to improve early cancer diagnosis in primary care: development of consensus guidelines. Oxford: University of Oxford, 2011.
48. McLachlan S, Mansell G, Sanders T, et al. Symptom perceptions and help-seeking behaviour prior to lung and colorectal cancer diagnoses: a qualitative study. *Family practice* 2015;32(5):568-77. doi: 10.1093/fampra/cmv048
49. Nicholson BD, Mant D, Neal RD, et al. International variation in adherence to referral guidelines for suspected cancer: a secondary analysis of survey data. *The British journal of general practice : the journal of the Royal College of General Practitioners* 2016 doi: 10.3399/bjgp16X683449
50. NICE. Suspected cancer: recognition and referral (NG12). NICE guideline: NICE, 2015.

For peer review only

COREQ Criteria Checklist

**Domain 1: Research team and reflexivity**<br><br>*Personal Characteristics*	Covered in article?	Page
1. Interviewer/facilitator Which author/s conducted the interview or focus group?	✓	8
2. Credentials What were the researcher's credentials? E.g. PhD, MD	✓	8, 1
3. Occupation What was their occupation at the time of the study?	✓	8
4. Gender Was the researcher male or female?	✓	8, 1
5. Experience and training: What experience or training did the researcher have?	✓	8, 1
Relationship with participants		
6. Relationship established: Was a relationship established prior to study commencement?	✓	9
7. Participant knowledge of the interviewer? What did the participants know about the researcher? e.g. personal goals, reasons for doing the research	✓	9
8. Interviewer characteristics What characteristics were reported about the interviewer/facilitator? e.g. Bias, assumptions, reasons and interests in the research topic.	✓	9
**Domain 2: study design**<br><br>*Theoretical framework*		
9. Methodological orientation and Theory What methodological orientation was stated to underpin the study? e.g. grounded theory, discourse analysis, ethnography, phenomenology, content analysis.	✓	9-10
Participant selection		
10. Sampling How were participants selected? e.g. purposive, convenience, consecutive, snowball	✓	8
11. Method of approach How were participants approached? e.g. face-to-face, telephone, mail, email	✓	8-9
12. Sample size How many participants were in the study?	✓	8
13. Non-participation How many people refused to participate or dropped out? Reasons?	No participants dropped out - our distributed recruitment methods means that we do not	

For peer review only

	know how many people saw information about the study	
Setting		
14. Setting of data collection Where was the data collected? e.g. home, clinic, workplace	✓	9
15. Presence of non-participants Was anyone else present besides the participants and researchers?	✓	9
16. Description of sample What are the important characteristics of the sample? e.g. demographic data, date	✓	8, 9
Data collection		
17. Interview guide Were questions, prompts, guides provided by the authors? Was it pilot tested?	✓	9
18. Repeat interviews Were repeat interviews carried out? If yes, how many?	NA	
19. Audio/visual recording Did the research use audio or visual recording to collect the data?	✓	9
20. Field notes Were field notes made during and/or after the interview or focus group?	Researchers kept their own field diaries with notes about the interviews – these were not intended for analysis	
21. Duration What was the duration of the interviews or focus group?	✓	9
22. Data saturation Was data saturation discussed?	✓	9
23. Transcripts returned Were transcripts returned to participants for comment and/or correction?	x	
**Domain 3: analysis and findings**<br><br>*Data analysis*		
24. Number of data coders How many data coders coded the data?	✓	9-10
25. Description of the coding tree Did authors provide a description of the coding tree?	n/a for this article	
26. Derivation of themes Were themes identified in advance or derived from the data?	✓	9-10
27. Software What software, if applicable, was used to manage the data?	✓	9
28. Participant checking Did participants provide feedback on the findings?	x (But PPI did)	10
Reporting		

For peer review only

29. Quotations presented Were participant quotations presented to illustrate the themes / findings? Was each quotation identified? e.g. participant number	✓	11-17
30. Data and findings consistent Was there consistency between the data presented and the findings?	✓	11-17
31. Clarity of major themes Were major themes clearly presented in the findings?	✓	11-17
32. Clarity of minor themes Is there a description of diverse cases or discussion of minor themes?	✓	11-17

For peer review only
